# Infliximab microencapsulation: an innovative approach for intra-articular administration of biologics in the management of rheumatoid arthritis—in vitro evaluation

**DOI:** 10.1007/s13346-023-01372-1

**Published:** 2023-06-09

**Authors:** Iván Lamela-Gómez, Lídia M. Gonçalves, António J. Almeida, Asteria Luzardo-Álvarez

**Affiliations:** 1 Department of Pharmacology, Pharmacy and Pharmaceutical Technology, Faculty of Sciences, Universidade de Santiago de Compostela, Campus Terra, 27002 Lugo, Spain; 2 Health Research Institute of Santiago de Compostela (IDIS), 15706 Santiago de Compostela, Spain; 3 Research Institute for Medicines (iMed.ULisboa), Faculty of Pharmacy, Universidade de Lisboa, Avenida Professor Gama Pinto, 1649-003 Lisbon, Portugal

**Keywords:** Infliximab, Microencapsulation, Rheumatoid arthritis, Intra-articular, Controlled release, Monoclonal antibodies

## Abstract

Microencapsulation of the therapeutical monoclonal antibody infliximab (INF) was investigated as an innovative approach to improve its stability and to achieve formulations with convenient features for intra-articular administration. Ultrasonic atomization (UA), a novel alternative to microencapsulate labile drugs, was compared with the conventional emulsion/evaporation method (Em/Ev) using biodegradable polymers, specifically Polyactive^®^ 1000PEOT70PBT30 [poly(ethylene-oxide-terephthalate)/poly(butylene-terephthalate); PEOT-PBT] and its polymeric blends with poly-(D, L-lactide-co-glycolide) (PLGA) RG502 and RG503 (PEOT-PBT:PLGA; 65:35). Six different formulations of spherical core–shell microcapsules were successfully developed and characterized. The UA method achieved a significantly higher encapsulation efficiency (69.7–80.25%) than Em/Ev (17.3–23.0%). Mean particle size, strongly determined by the microencapsulation method and to a lesser extent by polymeric composition, ranged from 26.6 to 49.9 µm for UA and 1.5–2.1 µm for Em/Ev. All formulations demonstrated sustained INF release in vitro for up to 24 days, with release rates modulated by polymeric composition and microencapsulation technique. Both methods preserved INF biological activity, with microencapsulated INF showing higher efficacy than commercial formulations at comparable doses regarding bioactive tumor necrosis factor-alpha (TNF-α) neutralization according to WEHI-13VAR bioassay. Microparticles’ biocompatibility and extensive internalization by THP-1-derived macrophages was demonstrated. Furthermore, high in vitro anti-inflammatory activity was achieved after treatment of THP-1 cells with INF-loaded microcapsules, significatively reducing in vitro production of TNF-α and interleucine-6 (Il-6).

**Graphical Abstract:**

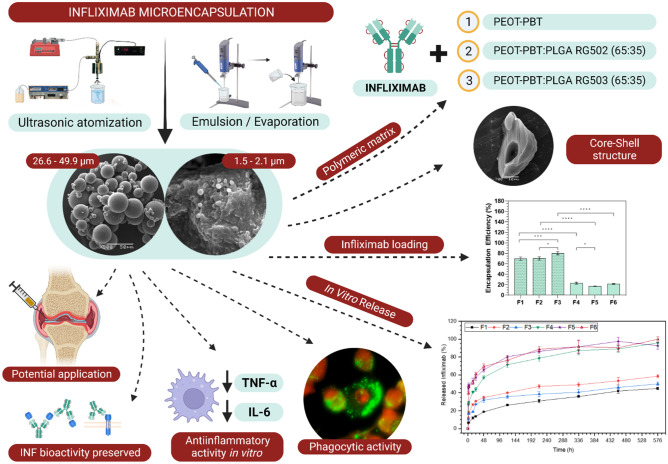

**Supplementary Information:**

The online version contains supplementary material available at 10.1007/s13346-023-01372-1.

## Introduction

The commercialization of disease-modifying biological drugs (DMARDs) with the authorization of infliximab (Remicade^®^) by the FDA in 1998 represented a milestone in the treatment of rheumatoid arthritis (RA) by offering a therapeutic alternative to patients’ refractory to conventional treatments. However, despite the extensive development of therapies based on blocking the tumor necrosis factor-alpha (TNF-α) signalling pathway and other mediators and cell types (interleukine-6, interleukine-1, T and B lymphocytes), the high cost of these treatments, in combination with their adverse effect profile, has prevented biological DMARDs from establishing as first-line treatment [1–4]. Local administration of biological DMARDs by intra-articular injection seems to be a promising approach to obtaining high concentrations of the monoclonal antibody at the site of action, increasing its efficacy while minimizing the potential systemic side effects. This local release would make sense in a context of localized inflammation of large joints that are easily accessible for injection, which in the case of RA corresponds to those situations in which, in routine clinical practice, it is necessary to resort to intra-articular injection of glucocorticoids to control pain and inflammation. Local treatment of RA is frequent either in the early stages after diagnosis before disease control is achieved or in patients with an active joint despite having achieved the goal of low activity with the background RA treatment. Likewise, it would be interesting in those less prevalent disease variants involving one or several localized joints, in monoarthritis, or even in other inflammatory joint pathologies such as osteoarthritis [4–6].

Although the local administration of anti-TNF-α antibodies has aroused great interest due to the high efficacy observed after systemic administration, their rapid clearance and short residence time in the joint cavity limited their use as long and medium-term therapy [7, 8]. The intra-articular administration of infliximab (INF) and other biological DMARDs has been reported, showing promising results in symptomatic relief and a potential delay of the disease progression [7–9], even though no multicentre clinical trial has been carried out to assess its efficacy after its intra-articular administration in solution. Initial findings from various authors indicate that locally administered biological DMARDs have demonstrated high efficacy, sometimes comparable to the local administration of glucocorticoids [10]. Therefore, in recent years, there has been a growing interest in developing drug delivery strategies that allow for the sustained release of biologics in the joint and its potential clinical applications [11–13].

A controlled infliximab delivery system with adequate properties to preserve its biological activity might obtain a prolonged INF release profile over time and achieve effective retention in the joint cavity. This delivery system should present the optimal characteristics to overcome the main limitations of biological DMARDs intra-articular administration. Microencapsulation, combined with an optimal polymeric matrix and formulation parameters, might be an adequate approach to reach those objectives [14]. Furthermore, Pradal et al. [15] observed in an in vivo mouse model of RA that particle size was a critical factor for the effective retention of particles in the highly permeable, inflamed joint cavity, establishing 10 µm as the minimum size to achieve the total retention of the formulation.

**Fig. 1:**
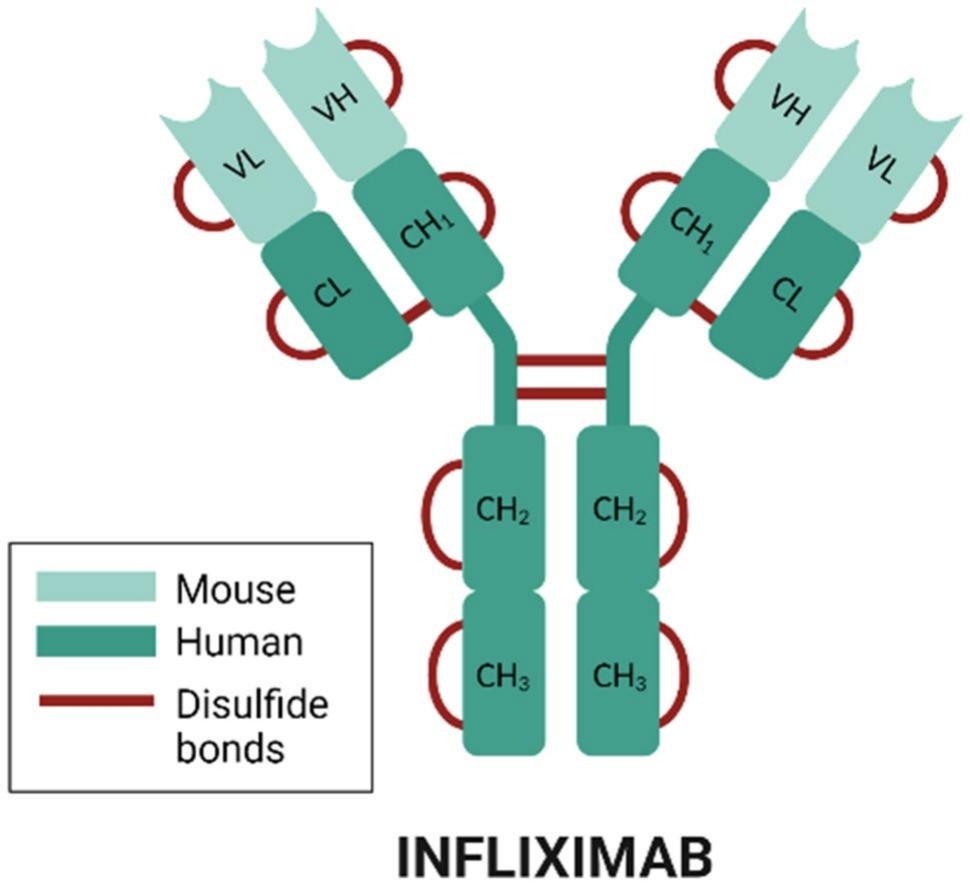
Schematic structure of infliximab. *Created with BioRender.com*

The main objective of this work is to develop a biodegradable microparticulate infliximab delivery system with potential application in the local intra-articular treatment of RA. Infliximab (Fig. 1) is a murine monoclonal antibody composed of the Fc region of a human immunoglobulin G (IgG 1; 75%) and a mouse variable region (25%) designed explicitly for binding to human TNF-α, presenting the ability to bind both soluble TNF and membrane TNF [16]. The inhibition of TNF-α by binding and forming stable complexes with INF has been shown to have high therapeutic efficacy, especially when administered in combination with methotrexate or other traditional DMARDs [17].

The microencapsulation of monoclonal antibodies such as INF implies a challenge since they have a high molecular weight (≈150 KDa), and it is necessary to preserve their high-order structure during the microencapsulation process. The production of microcapsules (MCs), depending on the technique and polymer used, involves a series of stress factors that can put this conformational stability at risk, such as contact with liquid–air interfaces, contact with organic solvents, mechanical stress during emulsification, and even contact with the hydrophobic domains of the polymeric matrix or the exposure to its degradation products during its degradation process [18, 19].

For the microencapsulation of INF, a hydrophilic poly(ethylene-oxide-terephthalate)/poly(butylene-terephthalate) block copolymer (PEOT-PBT; Polyactive^®^ 1000PEOT70PBT30) was selected, which has been shown to have excellent properties to preserve the stability of biomolecules and obtain a sustained release profile over time [20–22]. In addition, polymeric blends of PEOT-PBT with two varieties of poly-(D, L-lactide-*co*-glycolide) (PLGA RG502 and RG503) were used to investigate the polymeric composition’s effect on the microcapsules’ characteristics. As a technological approach for the microencapsulation of INF, coaxial ultrasonic atomization (UA) was employed and, comparatively, the widely investigated double emulsion (w/o/w)/solvent evaporation method (Em/Ev) [23, 24]. Coaxial ultrasonic atomization has suitable characteristics for the microencapsulation of labile molecules, such as monoclonal antibodies, since it allows us to obtain reservoir-type microcapsules under mild conditions, minimizing the exposure of proteins to adverse conditions. All developed formulations were characterized by their particle size distribution, drug entrapment, morphology, physicochemical properties, and microencapsulated infliximab stability. In addition, the in vitro cellular response of the INF-loaded formulations was evaluated in cell cultures of THP-1 monocyte-derived human macrophages.

## Materials and methods

Polyactive^®^ 1000PEOT70PBT30 multiblock copolymer with a 30 weight% of poly(ethylene-oxide-terephthalate) (PEOT; Mw = 1000 Da) and a 70 weight% of poly(butylene-terephthalate) (PBT) was purchased from PolyVation^®^ (Groningen, The Netherlands). Ester end-capped PLGA [poly-(D,L-lactide-*co*-glycolide)] copolymers with the same co-polymerization rate (lactide:glycolide) and different molecular weights, Resomer^®^ RG502 (50:50; Mw = 7000–17,000 Da) and Resomer^®^ RG503 (50:50; Mw = 24,000–38,000 Da), were obtained from Boehringer Ingelheim (Ingelheim, Germany). Infliximab (Remicade^®^) was obtained from Janssen Biologics (Leiden, The Netherlands). Coumarin 6, lipopolysaccharides from *E. coli* (LPS), propidium iodide (PI), PVA (poly (vinyl alcohol); Mw = 30,000–70,000 Da), Rose Bengal dye (RB), TNBS (2,4,6-trinitrobenzenosulfonic acid), and WST-1 cell proliferation assay were supplied by Sigma-Aldrich (Madrid, Spain). Dichloromethane, XTT sodium salt, and Resazurin sodium salt were purchased from PanReac AppliChem (Barcelona, Spain). Phenazine methosulfate (PMS) was supplied by Acros Organics (Geel, Belgium). All solvents were HPLC grade, whereas all other reagents were analytical grade.

### Preparation of microcapsules

Infliximab-loaded and empty microcapsules were fabricated using two different approaches: ultrasonic atomization (UA), as an innovative approach for microencapsulation of proteins and labile drugs, and a double emulsion/solvent evaporation method (Em/Ev) as one of the best-characterized and most widely explored microencapsulation techniques. Three different polymer compositions were used to obtain all microparticle formulations. Concisely, Polyactive^®^ 1000PEOT70PBT30 (PEOT-PBT) and its polymeric blends with two PLGA copolymers (Resomer^®^ RG502 and Resomer^®^ RG503) in accordance with Table 1.

**Table 1:** Different polymeric composition and microencapsulation techniques used to prepare the six formulations of microcapsules developed. Weight ratios of polymers are expressed as a percentage

**Formulation**	**PEOT-PBT**	**PLGA 502**	**PLGA 503**	**Technique**
**F1**	100%	0%	0%	UA
**F2**	65%	35%	0%	UA
**F3**	65%	0%	35%	UA
**F4**	100%	0%	0%	Em/Ev
**F5**	65%	35%	0%	Em/Ev
**F6**	65%	0%	35%	Em/Ev

A dual-feed coaxial ultrasonic nozzle (Sono-Tek Corp., Milton, USA) equipped with an ultrasonic generator operating at a fixed frequency (60 kHz) and variable power was employed to prepare the microcapsules (MCs). An infliximab solution (9 mg/ml) in 10 mM phosphate-buffered saline (PBS; pH 7.2) supplemented with 0.5% PVA (w/w) was infused through the inner channel at a flow rate of 0.3 ml/min, whereas a 4% (w/w) polymeric dispersion of PEOT-PBT, PEOT-PBT/PLGA 502, or PEOT-PBT/PLGA 503 (see Table 1) in CH_2_Cl_2_ was pumped through the outer channel at a constant flow rate of 1.5 ml/ml. A programmable syringe pump (NE-1000; New Era Pump Systems Inc, Farmingdale, USA) and a variable flow rate Merck-Hitachi L6000 pump (Merck-Hitachi, Tokyo, Japan) were utilized to feed the inner and the outer channel, respectively. The power supplied to the nozzle was set at 3.5 Watts. A stable, fine, small-drop spray was obtained and collected over a 3% (w/w) PVA stirring solution in PBS (100 mM, pH 8.1) supplemented with NaCl (45 mg/ml). Finally, the solvent was removed by rotaevaporation at 37 °C, and formulations were then isolated by filtration under pressure over a 0.8 µm regenerate cellulose membrane, washed twice with 100 ml of deionized water, and dried under vacuum.

Infliximab microencapsulation was also performed following a double emulsion/solvent evaporation technique. Briefly, 500 µl of an infliximab solution in PVA (2.5% w/v) was emulsified into 5 ml of a 6% (w/v) polymeric dispersion of PEOT-PBT, PEOT-PBT/PLGA 502, or PEOT-PBT/PLGA 503 (see Table 1) in CH_2_Cl_2_ for 3 min (Ultra-Turrax^®^ T10 basic, IKA, Staufen, Germany). Subsequently, the primary emulsion (w/o) was emulsified in PVA 1.25% (w/v) for 10 min at maximum speed using a Silverson L5M homogenizer (Silverson Machines Ltd., Chesham, UK), obtaining a w/o/w double emulsion which was maintained under continuous stirring for 4 h, at room temperature, to completely remove the organic solvent. Microcapsules were collected by centrifugation (5723 × *g* for 10 min, 4 °C; Allegra™ 64R Centrifuge, Beckman Coulter Inc., Brea, CA. USA), resuspended in purified water, and recollected by centrifugation twice for washing. Finally, MCs were redispersed into a 1% (w/v) solution of sucrose:trehalose (1:1) before freeze-drying (Christ Alpha 1–4, B. Braun Biotech International, Melsungen, Germany).

### Process yield and encapsulation efficiency

The amount of drug within the microcapsules was assessed following a modification of the method previously reported by Bezemer et al. [22]. This method is based on acid hydrolysis of the polymeric matrix and entrapped proteins. Once degraded and separated from polymer debris, protein concentration can be easily quantified using a method based on the reaction of the free amino groups in the amino acids with TNBS (2,4,6-trinitrobenzenesulfonic acid). Briefly, 25 mg of microcapsules was incubated with 1 ml of 6 M HCl for 48 h at 60 °C, then 6 ml of 1 M NaOH was added, and incubation was maintained in the same conditions for 24 h. The obtained suspension was then filtered through a 0.22-µm syringe filter, and infliximab concentration was determined using TNBS as follows: 50 µl of the sample was mixed with 125 µl of 1 M NaHCO_3_ buffer (pH 9) and 50 µl of 0.5% (w/v) TNBS aqueous solution in a 96-well plate. After 2 h of incubation in the dark, absorbance at 450 nm was measured using a microplate reader (Multiskan Ascent, Thermo Fisher Scientific, Waltham, MA, USA). All assays were performed in triplicate, and empty microcapsules were used as a reference. For quantification, a standard curve was obtained in the same conditions using pure infliximab standards to a final concentration between 5 and 160 µg/ml (*A*_450 nm_ =  − 0.0215 + 0.007 [infliximab]; *R*^2^ = 0.9999). Infliximab’s encapsulation efficiency (E.E.) was expressed as the percentage of drug within the microcapsules with respect to the theoretically loaded infliximab (Eq. 1). Detection (LOD) and quantification (LOQ) limits were set, respectively, at 1.96 and 5.94 µg/ml, accordingly to ICH Q2(R1) guidelines.

1 $$E.E.\left(\%\right)=\frac{Infliximab\;within\;microcapsules}{Theoretical\;loaded\;infliximab}\times100$$

The process yield of both microencapsulation techniques was calculated for all formulations prepared according to Eq. (2).

2 $$Process\;Yield \left(\mathrm{\%}\right)= \frac{Dry\;microcapsules\;weight}{Polymer\;weight+Drug\;weight} \times 100$$

### Particle size analysis

The microcapsule size distribution was determined by light laser diffraction according to a Fraunhofer diffraction model. Formulations prepared by UA were analyzed on a Mastersizer microparticle size analyzer (Malvern Instruments, Malvern, UK), while those prepared by Em/Ev were analyzed using a Mastersizer 2000 analyzer equipped with a Hydro2000S aqueous suspension sample dispersion module (Malvern Instruments, Malvern, UK). Briefly, a small amount of dry microcapsules in powder form was dispersed into purified water, and then samples were sonicated for 1 min before performing size analysis to disrupt aggregates, getting a fine suspension. All samples were analyzed in triplicate, and results were presented as volume-weighted size distribution, characterized by d(v,0.1), d(v,0.5), and d(v,0.9), which represent the diameter below which the 10%, 50%, and 90% of the size distribution lie, respectively. The span of the size distribution was calculated according to Eq. (3), and d(v,0.5) was used to express the mean particle size. Furthermore, volume-weighted (D[3, 4]) and surface-weighted (D[2, 3]) mean diameters are also presented.

3 $$Span= \frac{d\left(v,0.9\right)-d\left(v,0.1\right)}{d\left(v,0.5\right)}$$

### Scanning electron microscopy

The surface and inner morphology of the microcapsules were observed by scanning electron microscopy (SEM). Microcapsules were cross-sectioned to scan the inner structure, following a modified version of the method described by Kim et al. [25]. In short, representative samples of the MC formulations were dispersed in a small amount of epoxy resin (Reagent A; 5 Minute^®^ Epoxy, Devcon^®^, Solon, OH, USA) until a homogeneous suspension was formed. An equal amount of catalyst (reagent B) was added, and both components were mixed for 1 min to start the polymerization reaction. The mixture was allowed to stand for 2 h to allow the resin to harden. The resulting pellet was cross-sectioned with a razor blade, making several random cuts, thus sectioning some of the MCs embedded in the epoxy matrix. Samples of whole and cross-sectioned MCs were mounted on aluminum stubs covered with double-sided, adhesive, electrically conductive carbon tape and sputter-coated with gold/palladium (SDC 005 Sputter Coater, BAL-TEC GmbH, Schalksmühle, Germany). Afterwards, SEM micrographs were taken using a JEOL JSM-6360LV scanning electron microscope (JEOL Ltd., Tokyo, Japan) at an accelerating voltage of 15 kV and appropriate magnification.

### ζ potential

The zeta potential (ζ) of the microcapsules was assessed through electrophoretic light scattering (ELS) with a ζ potential analyzer (ZetaPlus, Brookhaven Instruments Corporation, New York, USA). Dry powder samples were resuspended into appropriate measurement buffers and sonicated for 30 s in mild conditions. Measurements were carried out at 25 °C at different pH and ionic strength conditions. Concisely, the ζ potential was determined in a suspension of MCs in both 1 mM KCl (pH 7) and a buffer composed of PBS (10 mM) and KCl (1 mM) adjusted to pH 7.4. All determinations were carried out in triplicate, and ten measurements were performed for each sample. Statistical analysis was performed using the GraphPad Prism^®^ software package (v.6.01, GraphPad Software, Inc., San Diego, CA, USA).

### Surface hydrophobicity determination

The influence of the polymeric composition of the microcapsules (Table 1) over their surface hydrophobicity was assessed by measuring the adsorption of Rose Bengal (RB) dye (4,5,6,7-tetrachloro-2′,4′,5′,7′-tetraiodofluorescein disodium salt; Sigma-Aldrich) onto the surface of microcapsules [26]. Uniform suspensions of microparticles (1 mg) were incubated with different concentrations of Rose Bengal (7.5 and 120 µg/ml) in an orbital shaker at 20 °C for 24 h (Thermomixer comfort, Eppendorf corporation, Hamburg, Germany). Then, samples were centrifuged at 23,900 RCF for 15 min, and the concentration of free RB in the supernatant was determined at 549 nm using an Evolution 60 s UV–Vis spectrophotometer (Thermo Fisher Scientific, Waltham, MA, USA) according to the following calibration curve: ABS_549_ =  − 0.005 + 0.1033[RB] (*R*^2^ = 0.9999). All experiments were performed in phosphate buffer saline (PBS) 100 mM at pH = 7.4. Due to the high affinity of the dye for Eppendorf tubes and pipette tips [26], control samples in the absence of MCs were maintained in the same conditions.

The amount of RB adsorbed to the surface of the MCs (*q*_*eq*_; µg/mg) in each of the samples was calculated according to Eq. (4), in which *C*_*o*_ represents the initial concentration of RB (µg/ml), *C*_*eq*_ the RB concentration at equilibrium in the MC samples (µg/ml), *C*_*eqRB*_ the RB concentration at equilibrium in the controls in the absence of MCs (µg/ml), *V* the volume of the RB solution (ml), and *m* the mass of MCs (mg).

4 $$q_{eq}=\left[\left(C_o-C_{eq}\right)-\left(C_o-C_{eqB}\right)\right]\cdot\frac Vm=\left(C_{eqB}-C_{eq}\right)\frac Vm$$

Experimental isotherms were analyzed according to both Langmuir [26] and Freundlich [27] adsorption models. Langmuir equation is shown below (Eq. 4), in which *C*_*eq*_ is the concentration of free RB at equilibrium (µg·ml^−1^), *q*_*eq*_ is the amount of dye adsorbed per milligram of MCs (µg·mg^−1^), *k*_*1*_ is the affinity constant of RB for microcapsules (ml·µg^−1^), and* k*_*2*_ is the maximum adsorption capacity of the monolayer (µg·mg^−1^). Experimental data were also fitted to the Freundlich adsorption model. Equation (5) describes the adsorption isotherm, in which *q*_*eq*_ is the amount of RB adsorbed to the surface of the microcapsules (µg/mg), *C*_*eq*_ is the equilibrium concentration of RB (µg/ml), KF is the Freundlich constant (µg/mg) which is related to the adsorption capacity of the surface, and 1/*n* is a dimensionless constant related to the adsorption intensity. Data were fitted to both models by non-linear regression using the least-squares method using OriginPro 2022 (v. 9.9.0.225, OriginLab Corporation, Northampton, MA, USA).

5 $${q}_{eq}= \frac{{k}_{1}{k}_{2}{C}_{eq}}{1+{k}_{1}{C}_{eq}}$$

6 $$q_{eq}=K_F\cdot C_{eq}^{1/n}$$

### Differential scanning calorimetry

Thermal properties of commercial polymers, infliximab-loaded microcapsules, and empty microcapsules were analyzed using differential scanning calorimetry (DSC). Samples weighing 1–5 mg were placed in non-hermetic aluminum pans (Tzero pan, model 901,683.901, TA instruments, New Castle, DE, USA) and subjected to calorimetric analysis in triplicate using a Q20 (v.24.7) Differential scanning calorimeter equipped with an RSC40 Refrigerated Cooling System (TA instruments, New Castle, DE, USA) previously calibrated using indium as standard. Thermograms were recorded between 5 and 250 °C at a heating rate of 10 °C/min, with an empty aluminum pan as the reference.

The thermal behavior of INF in solution was also investigated. A 10 mg/ml INF solution (16 µl) was sealed in an aluminum capsule and analyzed between 37 and 95 °C, with a temperature ramp of 1 °C/min. A reference capsule containing 16 µl of the buffer used in the INF solutions was used. Results were analyzed using TA Universal Analysis 2000 software (TA Instruments, New Castle, USA).

### Fourier transform infrared spectroscopy

Physicochemical interactions between infliximab and the polymeric matrix into the MC, as well as between PEOT-PBT and both PLGA varieties in their polymeric blends, were characterized by Fourier transform infrared spectroscopy (FTIR). Before analysis, dry samples (infliximab, PLGA 502, PLGA 503, PEOT-PBT, empty MCs, and INF-loaded MCs) were mixed with KBr in an appropriate ratio (1:30–1:100) and compressed to obtain KBr disks. Transmission spectra were recorded over 400–4000 cm^−1^ using a Vertex 70v FT-MIR spectrometer (Bruker Corporation, Billerica, MA, USA).

### In vitro delivery profile

The influence of polymeric composition and microencapsulation technique over the infliximab in vitro delivery profile was investigated. In vitro delivery assays were performed at 37 °C under *sink* conditions. In brief, 20 mg of dry MCs was accurately weighed in borosilicate vials, resuspended in 5 ml of PBS (10 mM, pH 7.4) supplemented with 0.02% sodium azide, and incubated under continuous orbital shaking (Unimax1010/Inkubator 1000 incubator; Heidolph Instruments GmbH & Co., Schwabach, Germany). At predetermined intervals, 1 ml samples were collected and replaced with a fresh releasing medium. Suspensions of empty MCs were maintained under the same conditions as negative controls to ensure no interference in INF determination due to polymer matrix degradation products. All experiments were performed in triplicate. Infliximab concentration in the samples was determined using a bicinchoninic acid (BCA) protein quantification kit (QuantiPro™ BCA Kit) following the manufacturer protocol. A calibration curve of INF was constructed in a concentration range between 0.5 and 20 µg/ml (${A}_{570 nm}= 0.0016+0.0643 \left[INF\right])$, exhibiting good linearity (*R*^2^ = 0.9999), and high sensibility (LOD = 0.21 µg/ml; LOQ = 0.66 µg/ml).

To prevent INF adsorption to the glass surface of borosilicate glass vials, they were pretreated with the siliconizing agent Sigmacote^®^ (Sigma-Aldrich, Madrid, Spain), a solution of chlorinated organopolysiloxane in heptane, which forms a microscopic layer covalently bonded to the glass when applied to the vials’ inner surface. This layer has hydrophobic properties, repelling water and preventing INF surface adsorption, thus minimizing the potential degradation of the drug.

### SDS-PAGE electrophoresis

The stability of infliximab’s primary structure after microencapsulation was assessed by SDS-PAGE electrophoresis under reducing and non-reducing conditions. Before analysis, the infliximab content of samples and standards was determined using a BCA protein assay kit (QuantiPro; Sigma-Aldrich), and concentrations were equalized. Afterwards, 10 µl of infliximab samples and standards (200 µg/ml) was mixed with 10 µl of Laemmli sample buffer containing or not 5% β-mercaptoethanol for reducing or non-reducing environment, respectively, and then heat denatured at 95 °C for 35 min. Subsequently, SDS-PAGE analysis was performed on a 13% T (Total concentration of acrylamide plus bis-acrylamide) resolving gel (1.5 M Tris–HCl, pH 8.8) with a 5% T (0.5 M Tris–HCl; pH 6.8) stacking gel at a constant voltage of 200 V for 35 min using a Mini-PROTEAN^®^ 3 cell equipped with a PowerPac™ HC Power Supply (Bio-Rad Laboratories, Hercules, CA, USA). For molecular weight determination, Precision Plus Dual Color Protein Standards were used (Bio-Rad). All analyses were carried out in triplicate. Finally, gels were stained with Coomassie Brilliant Blue R-250 (Bio-Rad), and images were then recorded with a GD1000 Axygen^®^ Gel Documentation System (Corning Inc, Corning, NY, USA). Further analysis was performed using GelAnalyzer 19.1 software (available at http://www.gelanalyzer.com).

### In vitro TNF-α bioactivity assay

The mouse fibrosarcoma cell line WEHI-13VAR (ATCC^®^ CLR-2148™) was purchased from ATCC (American Type Culture Collection, Manassas, VA, USA). Adherent cells were cultured in vitro in sterile 75 cm^2^ flasks at 37 °C under a 5% CO_2_ atmosphere with 95% relative humidity. The culture medium consisted of RPMI 1640 (Biological Industries; Cat. No. 01–104-1a) supplemented with thermally inactivated fetal bovine serum (FBS; 10%), D-glucose (25 mM), HEPES (10 mM), L-glutamine (2 mM), sodium pyruvate (1 mM), penicillin G (100 Units/ml), streptomycin (100 µg/ml), and neomycin (100 µg/ml). Cells were subcultured in a 1:6 ratio every 3–4 days once the cells reached around 80% confluency. All supplements used for cell culture were purchased from Biological Industries Ltd. (Cromwell, CT, USA).

TNF-α bioactivity assay is based on the high sensitivity of WEHI 164 mouse fibrosarcoma cell lines to human TNF-α secreted by activated monocytic cells, which results in cell lysis in a dose-dependent manner after exposure to the supernatant of LPS-activated human monocyte cultures. The development of anti-TNF-α therapeutic monoclonal antibodies has made bioactivity assays essential tools for indirectly assessing their bioactivity since their binding to TNF-α prevents it from exerting its biological action and, therefore, its toxicity on WEHI cells. These assays have become benchmark tests for characterizing such drugs, achieving the milestone of being recognized in the European Pharmacopoeia as a reference method for evaluating Infliximab’s biological activity [28].

The ability of INF-loaded microcapsules to neutralize TNF-α produced in vitro by THP-1 macrophages in response to their stimulation with LPS was investigated by biological activity assay using the TNF-α sensitive cell line, WEHI-13VAR [29]. THP-1 monocytes were seeded (10,000 cells/well) in sterile 96-well plates and differentiated into macrophages, as described in “THP-1 cell culture and differentiation into macrophages.” THP-1 cells were incubated (37 °C; 5% CO_2_; relative humidity 95%) with LPS (0.5 µg/ml) for 2 h to stimulate TNF-α production and then the culture medium was replaced by a fresh medium containing different concentrations of free INF (1.2–2400 ng/ml) or infliximab-loaded MCs from the six formulations developed (200 µg MCs/ml). After 6 h, the culture media containing the cumulate production of TNF-α was collected and centrifuged at 12,000 rpm for 5 min (10 °C; Hettich Universal 32 R) to eliminate possible cell debris or MC formulations. The supernatant was frozen at − 80 °C to perform the bioactivity assay as described below.

The WEHI-13VAR cells were grown until 70–75% of confluence before performing the TNF-α bioassay. Then, cells were seeded in clear bottom flat black 96-well plates at 20,000 cells/well density and incubated overnight. Once adhered, actinomycin D (500 ng/ml) was added and cultures were then incubated for 20 h with the supernatant of LPS-stimulated THP-1 macrophages, which was obtained after exposing the macrophages to all investigated treatments and conditions. Then, the culture medium was removed and the cell viability of WEHI-13VAR cells was determined by AlamarBlue^®^ assay. Serial fold dilutions of free INF were also incubated with LPS-stimulated THP-1 cell supernatant to establish the dose–response relationship and calculate the INF dose capable of inhibiting TNF-induced mortality by 50% (IC 50).

### THP-1 cell culture and differentiation into macrophages

THP-1 human monocytic cells (ATCC^®^ TIB-202™, American Type Culture Collection, Manassas, VA, USA) were cultured in RPMI 1640 medium (Biological Industries; Cat. No. 01–104-1A) supplemented with 10% heat-inactivated FBS, 2 mM L-alanyl-L-glutamine, 1 mM sodium pyruvate, 10 mM HEPES, 25 mM D-glucose (total concentration), and 1% of an antibiotic/antimycotic mixture (10,000 units/ml penicillin G, 10 mg/ml streptomycin, and 10 mg/ml neomycin). Cell culture was maintained at 37 °C, under a 5% CO_2_ atmosphere with 95% relative humidity, in 75 cm^2^ T-Flasks at a cell density between 2 and 8 × 10^5^ cells/ml. Culture media, FBS, and all supplements were purchased from Biological Industries (Cromwell, CT, USA).

THP-1 monocytic cells can be differentiated into monocytic-derived macrophages after exposure to phorbol 12-myristate 13-acetate (PMA), exhibiting a macrophage-like phenotype, as previously reported [30]. For differentiation, cells were seeded at a suitable density (1 – 5 × 10^5^ cells/ml) in a complete RPMI 1640 growth medium supplemented with 50 ng/ml PMA (Abcam, Cambridge, MA, USA) and incubated for 48 h. Subsequently, PMA-supplemented media was removed and replaced by a fresh medium, and THP-1-derived macrophages were then incubated for 24 h in the absence of PMA previously to perform any experiments. For PMA addition, the required amount of a PMA stock solution in DMSO (50 mg/ml) was diluted with a complete culture medium to a final concentration of 50 ng/ml. An equal amount of pure DMSO was employed as a negative control.

### Cytotoxicity assays

The cytocompatibility of all formulations developed was tested by XTT assay. The reagents for carrying out the assay were prepared in our laboratory, and the assay conditions were optimized for the THP-1 cell line once it had differentiated into macrophages. A XTT sodium salt solution (1 mg/ml) in phenol red free RPMI 1640 and a solution of phenazine methosulfate (PMS) in PBS (3 mg/ml) were prepared and stored at 80 °C. Immediately before performing the test, XTT and PMS solutions were mixed in a 1:400 ratio to obtain the activated XTT reagent. Briefly, 15,000 THP-1 cells were seeded in 96-well plates and allowed for differentiation in the presence of PMA for 48 h. Upon differentiation, THP-1-derived macrophages were treated with free infliximab (1.5–800 µg/ml), infliximab-loaded MCs, or empty MCs (100, 200, and 800 µg MCs/ml) and co-incubated for 24 h. Following incubation, 50 µl of activated XTT reagent was added to each well, and the plate was incubated at 37 °C, protected from light, for 4 h. Finally, the absorbance at 450 nm was determined, and the number of viable cells was calculated according to a calibration curve in the 234–15,000 cells/well range. Obtained results were expressed in terms of the percentage of cell viability with respect to the negative control (non-treated cells). Biocompatibility of formulations obtained by Em/Ev (F4, F5, F6) was also determined by WST-1 cell proliferation assay. Cells were culture, differentiated, and exposed to treatments in identical conditions as for the XTT assay, as described above, and subsequently WST-1 was performed according to the manufacturer’s protocol (Roche diagnostics, Germany).

Cell viability of WEHI-13VAR cells was measured using a Resazurin-based cytotoxicity assay [31] in which the non-fluorescent dye Resazurin was reduced by metabolically viable cells into Resorufin, a high fluorescence compound. A Resazurin sodium salt stock solution (0.15 mg/ml) was prepared in Ca^+2^ and Mg^+2^ free PBS and maintained in the dark at − 20 °C. To determine cell viability, 20 µl of the Resazurin stock solution was added per well, cell cultures were then incubated in a humidified atmosphere (5% CO_2_, 37 °C) for 3.5 h, and finally, resorufin produced by viable cells was measured fluorometrically using a microplate fluorimeter (*λ*_ex_/*λ*_em_ = 560/590 nm; Fluostar Optima, BMG Labtech, Ortenberg, Germany). The viable cell number was calculated according to a standard curve performed in the same conditions in the range between 100 and 20,000 cells/well. The cell viability index was expressed as the percentage of fluorescence intensity of each treatment group in relation to the untreated cell’s control.

### Phagocytosis assay

The phagocytic capacity of THP-1 macrophages in the presence of MC formulations was investigated both by quantitative microplate assay and by fluorescence light microscopy. Fluorescent-labelled microcapsules were prepared using two fabrication methods and three different polymer compositions as described above. Fluorescent labelling of microcapsules was performed by the addition of 2.33 µg of the high fluorescent lipophilic dye Coumarin 6 (stock solution 10 mg/ml in CH_2_Cl_2_) per milligram of polymer to the polymer dispersion in CH_2_Cl_2_ previously to microcapsules preparation.

THP-1-derived macrophages grown in black 96-well plates (50,000 cells/well) were treated with different concentrations of Coumarin 6–loaded microcapsules (2.5–40 µg/well), and fluorescence was measured (*λ*_ex_/*λ*_em_ = 485/520), and obtained values were set as 100% uptake reference values. Cell cultures were then incubated for 2.5 h, and the percentage of internalized or cell-associated microparticles was assessed by measuring fluorescence again after three washing steps with PBS (10 mM; pH 7.4) to remove not cell-associated microcapsules. Eight replicated were assayed for each sample, and untreated cells were used as a negative control. The percentage of MCs cell-associated was calculated based on fluorescence intensity relative to the reference value for 100% phagocytosis. For data analysis, ANOVA followed by Tukey’s post hoc analysis was performed (IBM^®^ SPSS^®^ Statistics v 24.0, IBM Corp., Armonk, NY, USA).

The phagocytic activity of THP-1 macrophages was also evaluated by fluorescence light microscopy. Cells were cultured and differentiated at a density of 50,000 cells/well, under the same conditions as in the microplate phagocytosis assay, and incubated with 20 µg/well of fluorescent particles for 2.5 h. Following incubation, cultures were washed in triplicate with PBS and fixed with a 3.7% formaldehyde solution in PBS for 15 min. After cell membrane permeabilization with 0.1% Triton™ X-100 in PBS, 50 µl/well of propidium iodide (PI) solution in PBS (40 µg/ml) was added, and the plate was incubated in the dark for 20 min. PI is a fluorescent intercalating agent that cannot cross the cell membrane, making it necessary to permeabilize it to allow its entry into the cell. Once it binds to the genetic material, it displaces its fluorescence spectrum towards longer wavelengths leading to a significant increase in its fluorescence intensity.

Stained cells were examined under a fluorescence microscope (Olympus IX71, Olympus Corp., Tokyo, Japan) with a filter commonly used for fluorescein observation, which allows us to simultaneously observe the MCs labelled with Coumarin 6 (green) and the cell nucleus stained with PI (red). Ten different images were randomly acquired for each sample (DP71 camera, Olympus Corp), and the obtained images were analyzed. The phagocytosis index was expressed as the percentage of macrophages that have phagocyted at least one microparticle. All experiments were performed in triplicate.

### TNF-α and Il-6 ELISA

The in vitro cellular response of THP-1 macrophages after exposure to the developed microparticle formulations was investigated. Concisely, the production of TNF-α and interleukine-6 (IL-6), two of the main proinflammatory mediators in RA, was quantified after the treatment of macrophages with free INF, INF-loaded MCs, and empty MCs.

THP-1 monocytes were seeded in 24-well plates at a cell density of 400,000 cells/ml (≈ 157,895 cells/cm^2^) and differentiated into macrophages following the protocol previously described. Once differentiated, the culture medium was replaced, and cells were stimulated with LPS (2 µg/ml) for 2 h before the addition of the treatments under investigation. Cells were co-incubated for 22 h, in the presence of LPS, with free infliximab (20–0.37 µg/ml) and two concentrations (200 µg/ml and 50 µg/ml) of empty (F1–F6) and INF-loaded MCs (F1–F6). As a negative control, non-LPS-stimulated cells were maintained without treatment to assess the basal production of the investigated cytokines. As a positive control, cells were stimulated with 2 µg/ml LPS.

The cumulative production of TNF-α and IL-6 by THP-1 cells under the different conditions investigated was determined in the culture supernatants using a sandwich enzyme-linked immunosorbent assay (ELISA). The 96-well ELISA plates were assembled using specific human TNF-α (ab213467) and Il-6 (ab246838) antibody pairs and reagents provided in the manufacturer’s recommended accessory kit (ab210905; Abcam, Cambridge, UK). Assays were performed accordingly to the manufacturer’s protocol. A calibration curve of recombinant TNF-α (15.6 and 1000 pg/ml) and Il-6 (3.9 and 250 pg/ml) was incorporated into each plate for quantification. Experimental data for the relationship between analyte concentration and absorbance (*λ* = 450 nm) were fitted to an adequate mathematical model. All samples and standards were tested in duplicate. Statistical analysis was performed by ANOVA followed by post hoc analysis by Tuckey’s test.

## Results and discussion

### Infliximab microencapsulation

**Fig. 2:**
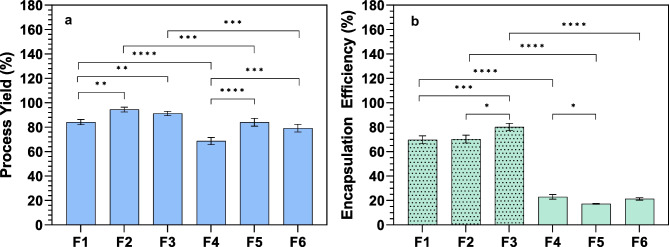
Process yield **a **and encapsulation efficiency **b **of formulations developed by ultrasonic atomization [F1 (PEOT-PBT), F2 (65:35 PEOT-PBT:PLGA RG502), F3 (65:35 PEOT-PBT:PLGA RG503)] and emulsion/evaporation [F4 (PEOT-PBT), F5 (65:35 PEOT-PB:PLGA RG502), F6 (65:35 PEOT-PBT:PLGA RG503)]. *(*p* ≤ 0.05); **(*p* ≤ 0.01); ***(*p* ≤ 0.001); ****(p ≤ 0.0001)

Infliximab was effectively microencapsulated using two methods and three polymeric compositions (Table 1). High process yield (P.Y) was achieved for all formulations, with statistically significant differences (*p* < 0.05) between techniques and higher P.Y for ultrasonic atomization (Fig. 2a). Regarding the influence of the polymeric composition, statistically significant differences were observed between PEOT-PBT formulations and those composed of its blends with PLGA within the same production procedure.

The microencapsulation method has been shown to have a statistically significant influence (*p* < 0.05) over infliximab’s encapsulation efficiency (E.E.) accordingly to ANOVA statistical analysis (Fig. 2b). Concisely, MCs prepared by UA exhibited high E.E (around 80% for the optimal formulation), whereas the emulsion evaporation (Em/Ev) technique resulted in low INF entrapment (around 20%). Although a statistically significant influence of polymeric composition over E.E. was also found, obtained results showed a stronger effect of the microencapsulation technique on INF entrapment.

The significative differences observed in E.E. depending on the microencapsulation procedure agreed with the high variability in entrapment rates previously reported for the encapsulation of monoclonal antibodies in polymeric nano- and microcapsules, ranging from 2 to above 80% [32–34]. Although emulsion-solvent evaporation methods have been extensively employed for microencapsulating hydrophilic and protein-based drugs, including monoclonal antibodies, this variability emphasizes the well-established challenge of achieving high encapsulation efficiencies for these drugs. The drug’s water solubility and the requirement for large volumes of the aqueous phase in the secondary emulsion contribute to protein diffusion into the external phase and subsequent drug loss.

During the optimization process of INF microencapsulation by UA, the pH value of the external phase of PVA was adjusted to the isoelectric point of infliximab to minimize its solubility in the continuous phase and reduce drug loss by diffusion. Moreover, the high concentration of PBS used for pH control and its supplementation with NaCl constitute a widely explored strategy in the microencapsulation of water-soluble molecules, which could partially explain the higher E.E. and slower release rate observed for these formulations compared to those prepared by emulsion evaporation [35]. In addition, the smaller particle size and higher surface area of the MCs prepared by Em/Ev (see Table 2) could have contributed to a greater diffusion of INF towards the external phase during emulsification and solvent evaporation steps, resulting in lower encapsulation efficiencies (see Fig. 2).

**Table 2:** Characteristic parameters of particle size distribution of MCs prepared by ultrasonic atomization [F1 (PEOT-PBT), F2 (65:35 PEOT-PBT:PLGA RG502), F3 (65:35 PEOT-PBT:PLGA RG503)] and emulsion/evaporation [F4 (PEOT-PBT), F5 (65:35 PEOT-PB:PLGA RG502), F6 (65:35 PEOT-PBT:PLGA RG503)]

	**d(v,0.1)** (µm)	**d(v,0.5)** (µm)	**d(v,0.9)** (µm)	**D **[3, 4] (µm)	**D **[2, 3] (µm)	**Span**
**F1**	16.74 ± 0.42	45.89 ± 0.45	97.58 ± 1.85	54.35 ± 0.93	18.16 ± 1.15	1.762 ± 0.016
**F2**	5.35 ± 0.12	26.61 ± 2.32	53.16 ± 0.08	31.28 ± 0.03	9.79 ± 0.10	1.711 ± 0.010
**F3**	18.90 ± 0.26	49.87 ± 0.10	93.76 ± 0.43	53.44 ± 0.09	16.02 ± 0.07	1.501 ± 0.012
**F4**	0.25 ± 0.02	2.09 ± 0.47	3.56 ± 0.68	2.15 ± 0.58	1.50 ± 0.88	1.354 ± 0.300
**F5**	0.23 ± 0.03	1.53 ± 0.27	3.03 ± 0.24	1.58 ± 0.23	0.68 ± 0.17	1.864 ± 0.289
**F6**	0.24 ± 0.02	1.71 ± 0.27	2.99 ± 0.28	1.77 ± 0.27	0.71 ± 0.13	1.625 ± 0.103

These results and those reported in the literature for the microencapsulation of similar macromolecules highlight the difficulty inherent in microencapsulating proteins of such high molecular weight. Furthermore, encapsulating labile macromolecules whose conformation is essential for their biological activity is challenging. Process conditions should be optimized to reach an equilibrium between appropriate formulation parameters to optimize the MC features and those that allow us to preserve the structural integrity of the microencapsulated molecule [18, 19]. Ultrasonic atomization, which allows us to obtain MCs in mild conditions and considerably reduces the protein mechanical stress compared to Em/Ev methods, seems to be an excellent approach to overcome the main limitations of monoclonal antibodies’ microencapsulation.

### Particle size analysis

Particle size distribution of all formulations developed was assessed by laser diffraction, and results were expressed in volume-weighted distribution (Table 2). Obtained results showed significant differences in size distribution depending on the microencapsulation technique and polymeric composition.

The microcapsules prepared by UA were characterized by a unimodal and polydisperse size distribution, with a mean diameter ranging between 26.6 and 49.9 µm. A significant influence of polymeric composition over mean particle size was observed, being significantly lower for formulation F2. Although the mechanisms involved in the process could not be completely elucidated, the size reduction observed in F2 seemed to be a consequence of the lower molecular weight of PLGA RG502 compared to PEOT-PBT and PLGA RG503; in fact, a direct relationship between the increase in the molecular weight of PLGA and an increase in particle size was previously reported [36]. Moreover, a lower viscosity was observed for the PEOT-PBT:PLGA RG502 blend dispersion in CH_2_Cl_2_ compared to PEOT-PBT and PEOT-PBT:PLGA RG503 dispersions. The influence of dispersion viscosity over droplet size obtained by UA, and its relationship with final particle size, depends on the inherent properties of each polymeric dispersion and formulation parameters. While some authors related a higher viscosity with an increase in droplet size [37], others related a higher viscosity to a longer residence time of the liquid on the nozzle surface, an increase in its temperature, and, as a consequence, a decrease in droplet size [23]. At constant power and flow rates, an increase in polymer concentration (and the subsequent increase in dispersion viscosity) led to an increase in particle size, as observed during the optimization of formulation parameters (data not shown). This effect was observed until a critical concentration value was reached, above which the nozzle could not generate an aerosol, leading to dropping and interrupting the atomization process. This critical concentration was established at 4.5% for the PEOT-PBT dispersion, while for the polymeric blends with PLGA RG503 and RG502, it was set respectively at 5% and 6%.

The size distribution of the formulations obtained by emulsion evaporation was analyzed once the solvent evaporation stage had finished before the purification process by centrifugation (Table 2). Once purified, the size distribution was examined again. A bimodal particle size distribution of a primary population in the micrometric range and a subpopulation smaller than 1 µm was obtained. The purification process eliminated the subpopulation of nanometric particles, obtaining a monomodal distribution for all the formulations investigated, with an average particle size between 1.5 and 2 µm. The average particle size obtained by this technique contrasts with that obtained by ultrasonic atomization (F1, F2, F3) using the same polymer composition. The observed size reduction can be attributed to a higher work input during the primary (w/o) and secondary (w/o/w) emulsification steps, compared to the mild emulsification conditions that characterize the coaxial ultrasonic atomization process, in which the secondary emulsion is generated by collecting the ultrasonic generated spray over a PVA solution under magnetic stirring [23]. Therapeutic monoclonal antibodies are susceptible to degradation or aggregation due to interfacial phenomena and shear forces generated during the high-speed emulsification processes used in emulsion evaporation techniques [19]. In light of these findings, those mild conditions make UA an excellent approach to microencapsulate bioactive biomolecules such as INF.

All the formulations developed presented an adequate size distribution for intra-articular administration. However, according to the results reported by Pradal et al. [15] after studying particle retention into the inflamed joint in an in vivo model of RA, microparticles with a diameter greater than 10 µm exhibited effective drug retention and higher residence time into the joint.

### Surface and inner morphology of the microcapsules

The surface and inner morphology of empty and INF-loaded MCs were investigated by scanning electron microscopy (SEM). Microparticulate formulations were embedded in an epoxy resin and cross-sectioned with a razor blade to observe their inner structure. Due to the limitations inherent to this method, only the internal structure of the formulations prepared by UA could be investigated since no sectioned particles could be observed in the formulations prepared by Em/Ev. This possibly resulted from their small particle size and aggregation in the solid state, which prevented them from adequately embedding in the resin matrix.

**Fig. 3:**
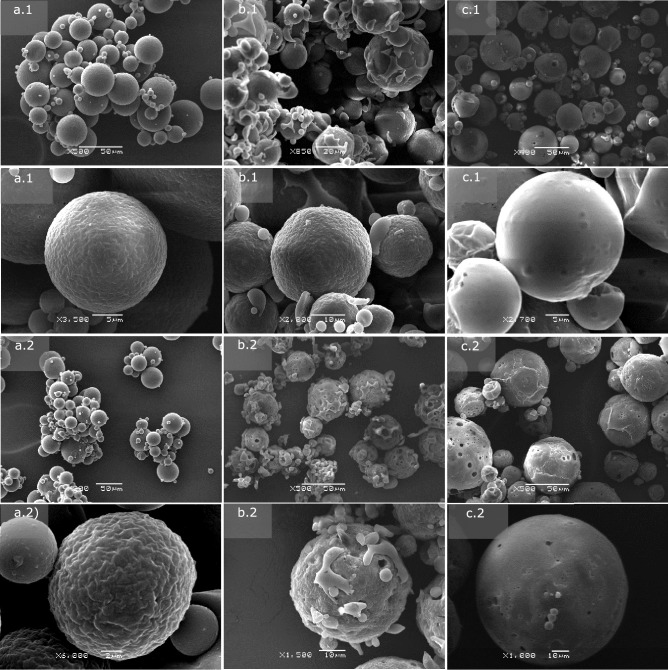
SEM micrographs of empty (1) and INF-loaded (2) microcapsules obtained by ultrasonic atomization: **a** F1 (PEOT-PBT), **b** F2 (65:35 PEOT-PBT:PLGA RG502), and **c** F3 (65:35 PEOT-PBT: PLGA RG503)

Microparticulate formulations prepared by coaxial ultrasonic atomization exhibited spherical particle size and a particle size distribution in agreement with the results obtained by laser diffraction. A low degree of aggregation was observed, and homogeneous suspensions in PBS and culture medium were prepared by simple manual shaking. SEM analysis of the formulations prepared by UA revealed differences in surface morphology depending on polymeric composition and the presence or absence of INF in the formulation (Fig. 3).

**Fig. 4:**
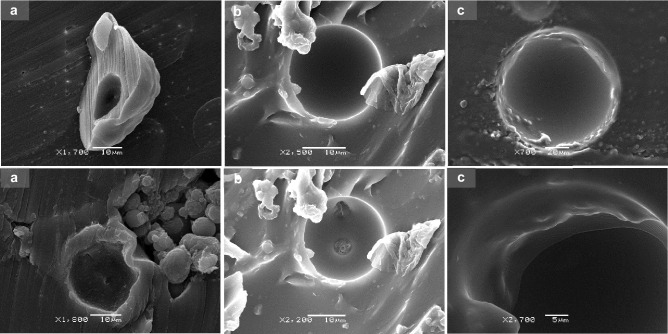
Cross-sectioned microcapsules obtained by ultrasonic atomization: **a** F1 (PEOT-PBT), **b** F2 (65:35 PEOT-PBT:PLGA RG502), and **c** F3 (65:35 PEOT-PBT:PLGA RG503)

Thus, formulation F1, prepared with 100% Polyactive^®^ 1000PEOT70PBT30 (Fig. 3a), was composed of spherical particles with a non-porous surface, which showed an irregular pattern at the microstructural level. Non-appreciable differences were observed between the surface morphology of the blank F1 MCs and those loaded with INF, although pores in small proportion were observed in the loaded formulations. Regarding the internal structure of the microparticles, the microphotographs of their cross section (Fig. 4a) confirmed that they were reservoir-type microcapsules composed of a thick polymeric shell surrounding a hollow core. One of the main advantages of coaxial ultrasonic atomization is the possibility of obtaining reservoir-type microcapsules using mild conditions compared to those used in double emulsion evaporation microencapsulation [23]. However, depending on the polymers used and the process conditions, other diverse internal structures of MCs have been obtained by other authors, ranging from mononuclear microcapsules, like those obtained in our case, to multinucleated or spongiform-like matrix particles [20, 38].

The observation of the MCs composed of 65% Polyactive^®^ and 35% PLGA RG502 (Fig. 3b), revealed the existence of spherical particles, on which small polymeric structures can be observed that seem to be fragments of a discontinuous cover that surrounds the particles. This type of morphology had not been previously observed during the preparation of MCs by this technique. Although the mechanisms involved in forming this type of morphology have not been elucidated, the results suggest a possible immiscibility of both polymers above a certain proportion, which leads to the segregation of part of the blend during the process of the solidification of the microparticles. On the other hand, the irregularity observed on the surface of the F2 MCs presented a pattern similar to that of the MCs composed only of PEOT-PBT, which supports the hypothesis that in the MCs composed of the PEOT-PBT:PLGA RG502 mixtures, a greater number of hydrophobic domains of the PEOT-PBT block copolymer were present at the surface level, which could explain the greater surface hydrophobicity observed for F2 compared to F3, as shown.

Regarding the effect of INF on the morphology of the MCs, superficial pores in some microcapsules were present (F1 formulation), as well as a greater surface irregularity. The observation of the internal morphology (Fig. 4b.1) confirmed that it is of reservoir-type microcapsules, with a single core surrounded by a dense polymer shell. Also, strong binding of the particles to the epoxy matrix was observed, making it challenging to observe the thickness of the coating.

To estimate the thickness of the shell (Fig. 4b), it was possible to examine the interior of the microcapsule at high magnification (× 24,000). It must clear up that first, the MCs show a high sensitivity to the electron beam used in the SEM, which gave rise to holes in the cover in the exposed area (Fig. 4b.2), and second, the resin matrix did not experience damage when subjected to the same observation conditions. The surface morphology of the microcapsules of formulation F3, composed of 65% PEOT-PBT and 35% PLGA RG503, showed relevant differences when incorporating INF in the MCs (Fig. 3c). While the empty formulations were characterized by a primarily smooth, non-porous surface with slight irregularities, the morphology of the INF-loaded microcapsules showed greater porosity and an irregular surface. This irregularity seems due to a thin, sometimes folded, or discontinuous covering around the particle, often located around the most porous regions. The composition of this structure is unknown but may be INF, found either internally, on the surface, or both. This could explain its absence in blank formulations and its presence near pores. Previous studies reported similar protein findings around pores after encapsulating BSA in PLGA [39].

On the other hand, it should be noted that this possible presence of INF on the surface contrasts with the high encapsulation efficiency observed for this formulation, which was the highest among the 6 formulations developed. The internal structure of the microcapsules (Fig. 4c) was similar to that observed in F1 and F2, confirming the mononuclear structure of the microcapsules. The internal surface of the MCs, as in the other formulations, did not show any pore, which indicates that these depressions were found at the surface level only, and, therefore, it was not established a direct connection with the nucleus of the particles once solidified.

**Fig. 5:**
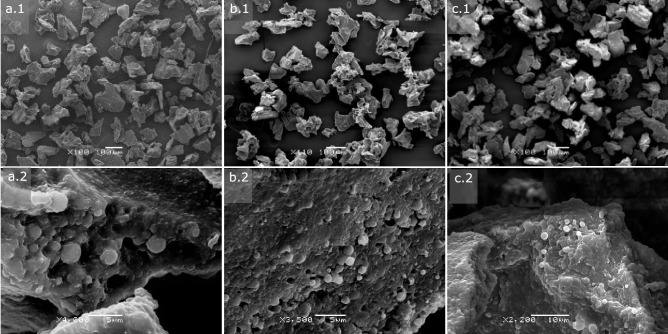
SEM micrographs of microparticulate formulations prepared by double emulsion/evaporation technique at low magnification (1) and close-up view (2): **a** F4 (PEOT-PBT), **b** F5 (65:35 PEOT-PBT:PLGA RG502), and **c** F6 (65:35 PEOT-PBT:PLGA RG503)

The surface morphology of the formulations prepared by the double emulsion-solvent evaporation technique was also investigated by SEM and the MC formulations were observed once they were lyophilized. The images obtained for the three investigated formulations (Fig. 5) showed that the lyophilized formulations were arranged in irregular aggregates of approximately 100 µm. The observation of the aggregate surface revealed that they were composed of spherical microparticles, possibly embedded in a trehalose matrix:sucrose (1:1) since the mixture of disaccharides represents around 25% (p/p) of the lyophilized formulations. The particle size observed in the 3 investigated formulations corresponded with the size distribution results obtained by laser diffraction. Regarding the surface morphology, the PEOT-PBT (F4) microcapsules showed a more irregular surface, similar to that observed in the larger MCs prepared by UA (F1), while those composed of the polymeric blends presented a smoother surface.

Despite the aggregation observed in the lyophilized formulations, preparing homogeneous suspensions in PBS and culture medium was possible using intense manual agitation and subsequent homogenization in an ultrasonic bath for 30 s. This behavior demonstrated the well-characterized anti-aggregating and cryoprotective effect of the disaccharides added to the formulation to avoid forming stable aggregates during lyophilization. In fact, during the optimization process of the formulations, it was observed that in the formulations prepared in the absence of anti-aggregant, stable aggregates were formed, whose average size was greater than 10 µm even after 30 min of homogenization in an ultrasonic bath. Therefore, adding sucrose and trehalose as cryoprotective agents positively impacted the suspension formulations’ properties.

In summary, it can be concluded that microencapsulation of INF by coaxial ultrasonic atomization using different polymer compositions (Table 1) resulted in three formulations with different size distributions and surface morphology (F1, F2, F3). At the level of internal morphology, all the formulations presented a microcapsule-type structure, with an internal core surrounded by a polymeric shell, which did not present pores on its inner face. Regarding the formulations obtained by emulsion evaporation (F4, F5, F6), microcapsules with a smaller average size and less polydispersity were obtained compared to those obtained for the formulations prepared by UA with the same polymeric composition. Regarding morphology, all the formulations prepared by emulsion evaporation are spherical microparticles that are arranged, forming aggregates (≈100 µm) together with the cryoprotectant matrix.

### ζ potential

**Fig. 6:**
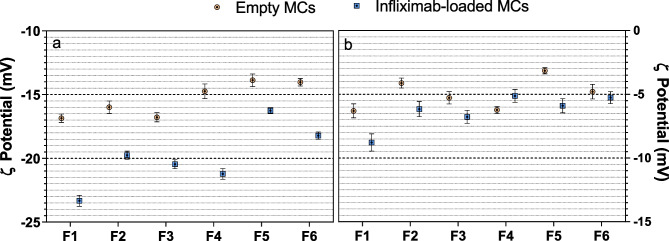
ζ Potential of empty and INF-loaded MCs prepared by UA [F1 (PEOT-PBT); F2 (65:35 PEOT-PBT:PLGA RG502); F3 (65:35 PEOT-PBT:PLGA RG503)] and Em/Ev [F4 (PEOT-PBT); F5 (65:35 PEOT-PBT:PLGA RG502); F6 (65:35 PEOT-PBT:PLGA RG503)] in different buffer: **a** KCl 1 mM; **b** KCl (1 mM), PBS (10 mM), pH = 7.4

The determination of ζ potential of the microparticulate formulations was carried out in a suspension of MCs in both 1 mM KCl (pH 7) (Fig. 6a) and a buffer composed of PBS (10 mM) and KCl (1 mM) adjusted to pH 7.4 (Fig. 6b). It should be noted that values of ζ potential obtained in different media should not be compared, due to significative difference in ionic strength and conductivity between both media. Regardless of the measurement conditions, all formulations exhibited a slightly negative surface charge.

According to the results obtained in both measurement conditions, the incorporation of INF in the MCs caused a displacement of the ζ potential towards more negative values. This effect was stronger in the formulations composed only of Polyactive^®^ (F1 and F4). Since the isoelectric point (pI) of the predominant species of INF is around 7.6, the antibody is expected to be positively charged under the measurement conditions. Moreover, in 1 mM KCl, the pH of the medium (pH ≈ 7) was lower than the pI of the most acidic species of INF (pI ≈ 7.3). Therefore, all the species of INF had a net positive charge under those conditions [40], so the decrease in ζ potential in the INF-loaded formulations could not be attributed to a direct effect of the presence of a significant amount of surface-adsorbed infliximab.

Different hypotheses could explain this modification in surface charge: first, protein-PVA interactions during microencapsulation could decrease ζ potential due to PVA adsorption on microcapsules’ surface. Negative acetate groups in PVA and INF-PVA interactions may contribute to this effect [39]. Second, from a different point of view, INF presence could alter polymer chain arrangement, impacting microcapsule surface charge. Proteins, including monoclonal antibodies, possess surfactant properties in drug delivery systems. Interactions between therapeutic antibodies and surfactants like PEG [poly(ethylene glycol)] have been observed, leading to antibody molecules replacing PEG at the liquid–air interface and reducing surface tension [41].

The most likely explanation for the observed surface charge changes in the MCs is the effect of INF on the polymeric matrix during the formation process. It should be noted that the more pronounced changes in formulations composed of Polyactive^®^ (F1 and F4), whose soft segment (PEOT) is structurally analogous to PEG, suggest an interaction between its interfacial properties and those of INF, influencing the polymer shell formation process. Even though statistically significant differences were found between the ζ potential of the different formulations developed (ANOVA; *p* < 0.05), these slight differences probably are not expected to affect the behavior of the microcapsules in terms of physiological response in an in vitro or in vivo environment.

### Surface hydrophobicity

The influence of polymeric composition on the surface hydrophobicity of MCs prepared by UA was assessed through the adsorption of the lipophilic dye Bengal Rose (BR). Experimental adsorption isotherms were fitted to Langmuir and Freundlich’s adsorption models. Experimental adsorption isotherms were fitted to both models (Table 3) according to the high values obtained for the adjusted determination coefficient (adj-*R*^2^). Nonetheless, a better goodness-of-fit was obtained for the Langmuir model, so it was selected for results interpretation according to the adsorption model’s estimated parameters (see the Supplementary Materials Section).

**Table 3:** Parameters of the Bengal Rose adsorption isotherms fitted to the Langmuir and Freundlich models [F1 (PEOT-PBT; UA), F2 (65:35 PEOT-PBT:PLGA RG502; UA), F3 (65:35 PEOT-PBT:PLGA RG503; UA)]

	**F1**	**F2**	**F3**
**Langmuir**			
* K*_1_	0.1396 ± 0.0271	0.0879 ± 0.0095	0.04561 ± 0.00921
* K*_2_	126.1058 ± 5.5988	120.6611 ± 3.6687	94.8141 ± 6.9248
* R*^2^	0.99922	0.99964	0.99793
Adj-*R*^2^	0.99902	0.99955	0.99741
**Freundlich**			
* K*_F_	24.5943 ± 3.1129	19.3223 ± 1.6303	10.3501 ± 0.7806
1/*n*	0.4270 ± 0.0425	0.4412 ± 0.0265	0.4756 ± 0.0211
* R*^2^	0.98149	0.99329	0.99619
Adj-*R*^2^	0.97686	0.99161	0.99524

Langmuir and Freundlich adsorption isotherms obtained for the three investigated formulations showed that BR has a different affinity for the surface of the MCs depending on its polymeric composition (F1 > F2 > F3). The value of the Langmuir affinity constant (*k*_*1*_) is directly related to the hydrophobicity of the surface, thus evidencing the greater hydrophobicity of F1, composed of 100% PEOT-PBT compared to the MCs composed of their polymeric blends with PLGA RG502 (F2) and PLGA RG503 (F3). Regarding the surface hydrophobicity of F2 and F3, the values obtained for *k1* suggested that the PEOT-PBT/PLGA RG502 (F2) MCs presented a more hydrophobic surface than those made by the PEOT-PBT/PLGA RG503 (F3). That observation was not expected, considering the relative molecular weight and hydrophobicity of both varieties of PLGA, being significantly greater for PLGA RG503 (24,000–38,000 Da) compared to PLGA RG502 (7000 and 17,000 Da). A hypothesis that may partially explain this phenomenon is the different arrangement of both PLGA copolymers in their blends with PEOT-PBT, resulting in a higher exposition of hydrophobic domains at surface level in the MCs composed of PEOT-PBT:PLGA 502 (F2). The estimated values for *k*_*2*_, which represents the maximum adsorption capacity of BR to the surface of the MCs (µg/mg), followed the same trend as *k*_1_ (F1 > F2 > F3), although the difference between F1 and F2 in terms of maximum capacity (*k*_*2*_) was lower compared to the differences observed in terms of affinity (*k*_*1*_). This observation could be explained by the differences in the formulation’s specific surface, which is probably higher for F2 due to its smallest particle size.

Regarding the Freundlich model, the parameter 1/*n* is inversely related to the affinity of the adsorbate (BR) for the surface of the adsorbent (MCs), and *K*_*F*_ is directly related to the adsorption capacity. Surface hydrophobicity results according to the Freundlich model (Table 3) agreed with the conclusions reached using the Langmuir model. Values obtained for both the Freundlich constant (F1 > F2 > F3) and 1/*n* constant (F3 > F2 > F1) suggest that the MCs composed only of PEOT-PBT showed the highest surface hydrophobicity, followed, respectively, by the formulations prepared with their blends with PLGA RG502 and PLGA RG503, which reinforces the conclusions reached previously.

In conclusion, obtaining microcapsules by ultrasonic atomization using only the Polyactive^®^ block copolymer led to MCs with a higher surface hydrophobicity than those formulations prepared with their polymeric blends with different varieties of PLGA. Since the structure of Polyactive^®^ consists of two well-differentiated segments whose hydrophobicity differs considerably, the results suggest that during the formation of the MCs, a high percentage of hydrophobic domains was oriented towards the outside of the particles. On the other hand, the greater surface hydrophobicity of the MCs composed of the Polyactive^®^/Resomer^®^ RG502 blend compared to those composed of Polyactive^®^/Resomer^®^ RG503 suggested a different behavior of both blends during the microencapsulation process. A plausible hypothesis to explain those results might be the presence of a more significant amount of PEOT-PBT on the surface of this formulation. Nonetheless, additional research should be performed to elucidate the mechanisms involved in obtaining microcapsules composed of PEOT-PBT and its polymeric blends with PLGA.

### Differential scanning calorimetry

DSC analysis has been reported as a suitable tool to assess antibody conformation and domain folding integrity. DSC thermogram of the commercial formulation of INF (Remicade^®^) in an aqueous solution showed the characteristic transition of F_c_ and F_ab_ regions of the antibody, as previously reported [42] (see Supplementary Materials). However, the DSC studies performed using extracted INF from microcapsules did not lead to conclusive results. The marketed INF formulation was used as a reference and diluted to the same extent as the released samples, which did not show any detectable characteristic transition for INF. Therefore, it would be necessary to use more precise and sensitive DSC equipment than the conventional system used in this work, specifically when using so low-concentrated antibody solutions.

DSC profiles of pure polymers (Polyactive^®^ 1000PEOT70PBT30, Resomer^®^ RG503, and Resomer^®^ RG502) and blank and INF-loaded microcapsules were also registered (see Supplementary Materials) to determine whether interactions between the drug and the polymeric matrix occurred or even between Polyactive^®^ and the two varieties of PLGA.

Resomer^®^ RG502 thermogram was characterized by an endothermic transition (48.5 °C) indicative of the relaxation peak after the glass transition temperature (*T*_g_ ≈ 42–46 °C) of the amorphous polymer [43]. The thermal profile of pure Resomer^®^ RG503 displayed an endothermic event at 55 °C corresponding to the structural relaxation following the *T*_g_ of this PLGA (≈ 40–65 °C), which has been demonstrated to increase with the molecular weight of the PLGA [44].

According to the literature, the thermal analysis of PEOT-PBT copolymers is characterized by the transitions of both segments as a consequence of the micro-separation of phases resulting in a heavy segment (mainly PBT) and a soft domain (mostly PEOT), probably owing to the poor compatibility between the two copolymer blocks [45, 46]. The DSC profile of Polyactive^®^ 1000PEOT70PBT30 was characterized by 3 endothermic transitions, one for the PEOT segment and two for the PBT block. The glass transition temperature (*T*_*g*_) of the PEOT phase was detected at 49 °C, whereas the characteristic melting peak of PEOT (*T*_m_ ≈ 3.2 °C) was not detected as it occurs at a lower temperature than the range studied [47]. A low-intensity endothermic event occurred at 40.5 °C, which may coincide with the PBT segment’s glass transition temperature, as described by other authors. However, it is usually not detected in polymers with such a low proportion of PBT (30%), ranging between 26 and 54 °C depending on the percentage of PBT in the copolymer (*T*_g_ of homopolymer ≈ 56 °C). A wide endothermic band with a maximum of 162.3 °C (ΔHm = 10.97 J/g) was identified as the characteristic melting point of the PBT segment [45–48].

DSC analysis of PEOT-PBT microcapsules fabricated using ultrasonic atomization (F1) and emulsion/evaporation (F4) exhibited the characteristic bands of both copolymer segments. No noticeable differences were detected between thermograms of blank and INF-loaded formulations, which could be a consequence of the low sensitivity limit of the DSC technique combined with the low proportion of INF within the microcapsules (2.4–2.8%). In both formulations was detected a low-intensity transition around 40 °C due to the *T*_g_ of the PBT segment. Formulation F1 exhibited a single wide melting point (PBT segment) at 161–163 °C, similar to the pure polymer (*T*_m_ ≈ 161 °C), while F4 showed a slight shift to 170–175 °C, possibly due to changes in the PBT segment’s crystalline structure as a consequence of the differences between the microencapsulation technique or, even more, the solvent evaporation rate during the preparation of each formulation [45–48]. These results confirmed that the PEOT-PBT copolymer preserved its semicrystalline structure after microencapsulation. These results confirm that the semicrystalline structure of the PEOT-PBT copolymer was preserved after microencapsulation. Additionally, INF incorporation into the microcapsules did not result in any notable changes in their thermal properties compared to the blank formulations.

Thermograms of PEOT-PBT:PLGA RG502 (65:35) formulations (F2, F5) showed characteristic thermal transitions for both polymers. A sharp endothermic peak (48–52 °C) indicated relaxation following PLGA RG502’s glass transition temperature (*T*_g_ ≈ 42–46 °C) [43], followed by a wide band around 162 °C characteristic of the *T*_m_ of the PEOT-PBT segment, demonstrating its disordered crystalline phase [46–48]. The observation of the characteristic bands for both polymers in the MCs’ thermograms at very close temperatures than those observed for pure polymers evidenced that polymers were immiscible or miscible in low proportion. These findings support the hypothesis that the polymeric coating of the microcapsules consisted of a simple blend of both polymers [49].

DSC profiles of microcapsules made from PEOT-PBT:PLGA RG503 (65:35) blend (F3, F6) revealed an endothermic peak at 53–55 °C, which corresponds to the relaxation event after the Tg of PLGA RG503, suggesting that the preparation process did not affect the polymer’s glass transition temperature compared to the pure polymer [44]. Nevertheless, the melting point of the PBT segment of Polyactive^®^ (*T*_m_ ≈ 162 °C) could not be detected. In contrast, we observed this peak in the MCs composed of PEOT-PBT mixed at the same proportion with PLGA RG502 (F2, F5). These findings suggest that the polymers may have exhibited higher miscibility in the blend or, even more, partial or complete solubilization of PBT in the PLGA matrix. The transition events of the PEOT segment were not detected because they are out of the temperature range of these studies (*T*_g_ =  − 50 °C; *T*_m_ = 6 °C) [45–49]. Further detailed calorimetric studies seemed necessary to investigate how these thermal findings could affect aspects such as, for example, the release behavior and the degradation rate of the polymer and, therefore, to the features of these microcapsules as drug delivery systems.

### Fourier transform infrared spectroscopy

Transmission FTIR spectra of free INF, INF-loaded MCs, and empty MCs were recorded to investigate possible interactions between the two shell-forming polymers and between them and INF. Also, pure polymers were analyzed. Full spectrums were displayed and analyzed in detail in the supplementary materials section.

Infliximab’s infrared spectrum was characterized by the bands corresponding to the amide group’s vibrational modes in the peptide bond. These included amide I (1644 cm^−1^), amide II (1542 cm^−1^ and 1456 cm^−1^), and amide III (1268 cm^−1^). Additionally, intense broadband at 3395 cm^−1^ (N–H stretch) was detected, followed by a lower-intensity triplet (2840–2955 cm^−1^) characteristic of CH_2_ and CH_3_ groups. These results are consistent with previous studies [50].

The FTIR spectrum of Polyactive^®^ 1000PEOT70PBT30 exhibited characteristic bands related to the functional groups in both copolymer segments, agreeing with previous reports on similar poly(ether/ester) block copolymers composed of PEOT and PBT [46, 51]. Specifically, high-intensity bands related to the terephthalate units were registered at 1720.1 cm^−1^ and 1272.9 cm^−1^ (aromatic C = O and C-O stretch), as well as other less intense bands at 1616.8 cm^−1^ (aromatic C–C stretch), 1027.8 cm^−1^, and 730.9 cm^−1^ (aromatic C-H bending). Related to the soft segment (PEOT), bands for aliphatic ethers at 1108.2 cm^−1^ and 872.7 cm^−1^ (asymmetric and symmetric C–O–C stretch) and broadband between 3000 and 2800 cm^−1^ for aliphatic CH_2_ (C-H symmetric and asymmetric vibration modes) were identified. Additionally, low-intensity broadband (3320 and 3580 cm^−1^) was recorded, possibly related to hydroxyl O–H stretch (3320 and 3580 cm^−1^), supported by a small band at 620 cm^−1^ (Hydroxyl O–H bending). While the copolymer structure lacks free hydroxyl groups, these low-intensity bands may be attributed to residual monomers, impurities, or degradation products present in the commercial polymer in small proportions.

The infrared spectrum of Resomer^®^ RG502 and RG503 was characterized by the distinctive bands of the functional groups found in the PLGA copolymers. Resomer^®^ RG502 spectrum displayed an intense band at 1759.8 cm^−1^ (ester C = O stretch) and lower-intensity bands at 2999.9 cm^−1^ (CH2 bending), 1426.4 cm^−1^ (CH stretch), and 1179.1 cm^−1^ and 1094.6 cm^−1^ (C-O stretch), consistent with previous findings [52]. Resomer^®^ RG503 displayed a similar spectrum with a prominent 1760.1 cm^−1^ band (ester C = O stretching), a low-intensity doublet at 2952.9 cm^−1^ and 2998.7 cm^−1^ (C-H stretching), as well as bands at 1425.5 cm^−1^ (C-H stretching), 1093.4 cm^−1^ and 1183.6 cm^−1^ (C-O stretching). The absence of free carboxyl groups (due to esterification) in these end-capped types of PLGA resulted in the lack of noticeable bands in the characteristic range of vibrational modes for OH groups (3200–3500 cm^−1^) [52].

The FTIR spectra of empty microcapsules (MCs) were analyzed, revealing characteristic bands of the polymers in their composition. No significant shifts in frequency or detection of new functional group bands were observed, suggesting no chemical reactions occurred between the components during the microencapsulation process. Those findings suggest that the polymeric matrix is a simple mixture of PEOT-PBT and PLGA RG502 or RG503. It is essential to highlight that the FTIR spectra of INF-loaded microparticles showed no significant differences from empty MCs, as no characteristic INF bands were detected. This was probably due to the method’s lack of sensitivity and a low proportion of INF (2.4–2.8%) in the polymer matrix.

When comparing the infrared spectra of the commercial PEOT-PBT copolymer and empty MCs made of PEOT-PBT or its polymeric blends with PLGA, it was found that the hydroxyl group bands (3320–3580 cm^−1^ and 620 cm^−1^) and low-intensity bands at 1662.3 and 1616.8 cm^−1^ observed in the spectrum of commercial Polyactive^®^ were absent. These bands around 1600 cm^−1^, related to the polymerization degree of the PBT segment [48], could be attributed to the presence of monomers, oligomers, or lower molecular weight impurities in the commercial polymer, which could have diffused into the aqueous phase during microencapsulation due to the dissolution and reprecipitation of the polymer, explaining its absence in the microcapsules. Although these bands were not theoretically expected according to the chemical structure of the polymer, they could be characteristic of their monomers or structurally similar compounds [48, 53].

The spectra of formulations made of PEOT-PBT polymeric blends with PLGA RG502 (F2, F5) and RG503 (F3, F6) did not show notable differences in band frequencies, as was expected due to the similar structure of both PLGA copolymers, which only differ in molecular weight. The peaks corresponding to the C = O tensions of the ester groups in PEOT-PBT (1720 cm^−1^) and PLGA (1760 cm^−1^) were identified in all formulations. Due to the greater susceptibility of this functional group to hydrolysis, the primary degradation route of both polymers, the identification of its characteristic bands confirmed that the polymeric matrix did not undergo significant degradation during the production and isolation of the MCs [51, 52].

### In vitro delivery profile

**Fig. 7:**
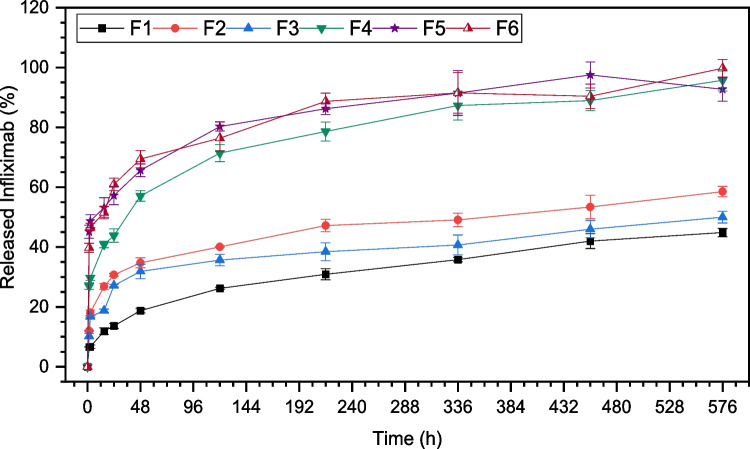
Infliximab in vitro delivery profile from microcapsules made by UA [F1 (PEOT-PBT); F2 (65:35 PEOT-PBT:PLGA RG502); F3 (65:35 PEOT-PBT:PLGA RG503)] and Em/Ev [F4 (PEOT-PBT); F5 (65:35 PEOT-PBT:PLGA RG502); F6 (65:35 PEOT-PBT:PLGA RG503)]. 37 °C; PBS buffer (10 mM, pH 7.4)

The in vitro release of INF from all microparticulate formulations developed was investigated at 37 °C in PBS (10 mM, pH 7.4) to assess the influence of the polymeric composition and the microencapsulation technique on the infliximab delivery profile (Fig. 7), evidencing that the polymeric composition and the microencapsulation technique greatly impacted the INF release rate.

The microcapsules composed of PEOT-PBT showed a lower release rate and initial release than the formulations prepared by the same technique and composed of PEOT-PBT:PLGA blends. The F1 formulation, prepared by UA, exhibited an initial release of 6.6% in the first 1.5 h, followed by a sustained release of INF over time that reached 44.9% of INF released after 576 h (24 days). On the other hand, formulation F4, prepared by emulsion evaporation, led to a higher initial release (26.9%), followed by a sustained release profile for 24 days, when 95.7% of its content had been released. Concerning the interpretation of results, the formulations prepared by emulsion evaporation have shown a significantly lower encapsulation efficiency than that obtained by ultrasonic atomization. Even though formulations prepared by emulsion evaporation exhibited a higher release rate, these differences can be magnified when representing the results in terms of the percentage of drug released (Fig. 7), being smaller in terms of drug released per unit of time, so the main difference lies in the maximum time during which sustained release of the drug can be achieved.

Although PEOT-PBT copolymers have not been widely used in the development of nano- and microparticles for controlled drug release, the advantages of these block copolymers in protein microencapsulation, their ability to preserve their biological activity, and the mechanisms involved in their release from this type of system have been investigated in depth [22, 54, 55]. Infliximab in vitro release profile from MCs composted by PEOT-PBT exhibited similar characteristics to those obtained by other authors for BSA as a model protein when using intermediate water/amount ratios. Hence, it is likely that formulation F1, which showed the lowest release rate, did not completely release the total amount of microencapsulated drug [54].

Microcapsules made of the polymeric blends between PEOT-PBT and PLGA RG502 and RG503 exhibited an increase in the initial release of INF compared to formulations composed of PEOT-PBT and prepared by the same technique. In addition, it is well known that one of the main limitations of PLGA for microencapsulation and release of macromolecules is the high initial burst release [32, 56]. Furthermore, the amount of drug initially released when MCs are suspended in aqueous media and particle size have been inversely related [57], which agrees with the higher initial release observed for formulations prepared by emulsion evaporation than those obtained by UA. However, no significant differences were observed between the INF release profiles of the MCs prepared by UA and composed of PEOT-PBT:PLGA RG502 and those prepared with the mixture PEOT-PBT:PLGA RG503, which could be due to the low proportion of these in the polymeric matrix (35%) compared to PEOT-PBT (65%). The incorporation of PLGA RG503 into the polymeric shell led to a release profile similar to the one observed for MCs composed of the blend with PLGA RG502 during the first 48 h, followed by a slower release rate. Concerning the formulations prepared by emulsion evaporation, no remarkable differences were observed between the INF release profiles from the MCs prepared with the PEOT-PBT:PLGA (65:35) blends. In broad terms, for the MCs prepared using the same technique, the release profile from the formulations composed only of PEOT-PBT led to the lowest initial burst and release rate of INF, which increased when PLGA was incorporated into the polymeric matrix.

In summary, all the microparticulate formulations developed showed the ability to obtain a sustained release of infliximab over time, and the release rate could be modulated depending on the polymeric matrix composition and the technological approach used for microencapsulation.

### Structural stability of infliximab

The structural integrity of commercial infliximab (Remicade^®^), pure INF previously atomized through the ultrasonic nozzle, and INF released from the microcapsules, was investigated by SDS-PAGE electrophoresis under reducing and non-reducing conditions. Stained gels were analyzed using the GelAnalyzer software package, and the approximate molecular weight of the bands was calculated according to a calibration curve obtained from the relative position (Rf) of the molecular weight standards bands (gel images were displayed in the Supplementary Materials Section). The calibration curve was successfully fitted to an exponential model for the standards analyzed in gel A (*R*^2^ = 0.9880) and gel B (*R*^2^ = 0.9960). Infliximab atomized through the ultrasonic nozzle, and all samples of INF extracted from the microcapsules exhibited two main bands at approximately 50 and 25 KDa, attributable to the heavy and light chains of INF, which presents the typical structure of IgG 1 isotype antibodies [58]. No significant differences were found between the band pattern obtained for the INF samples and the INF standard (Remicade^®^).

Concerning the analysis of INF extracted from the formulations prepared by UA (gel B), two low-intensity bands were observed around 165 and 213 KDa, which could be attributed either to a low percentage of aggregated antibody or to a small fraction of non-reduced INF. Furthermore, it is essential to highlight that these bands were also observed for the INF standard, so these do not seem to be a consequence of structural alterations induced by the microencapsulation process. In this sense, other authors obtained similar results after the extraction of INF from PLGA MCs prepared by emulsion evaporation and phase separation [32].

The INF standard and the antibody extracted from the microcapsules were also analyzed under non-reducing conditions. The results of the analysis did not give rise to differences in the bands observed in the commercial pattern and the samples of INF released from the formulations, with a band of greater intensity appearing that corresponds to the molecular weight of INF (≈150 KDa). Likewise, some lower-intensity bands were observed close to the molecular weight of the light and heavy chains, indicating a low proportion of fractionated antibodies. Since this technique is not quantitative, it was impossible to accurately estimate the percentage representing the fractionated antibody amount. However, in terms of optical density, no differences were observed between the relative intensity of the bands corresponding to the samples extracted from the microcapsules and those observed for the commercial formulation.

In light of the obtained results, Infliximab’s primary structure was preserved after the microencapsulation process, maintaining the characteristic IgG structure, composed of two heavy chains and two light chains linked by disulfide bonds. However, it is important to emphasize the need to perform further studies to assess the impact of the microencapsulation process on the secondary and tertiary structure of infliximab, which is essential to its biological activity.

### Biological activity of microencapsulated INF

The capacity of microencapsulated INF to neutralize TNF-α bioactivity was investigated in vitro in LPS-stimulated THP-1-derived macrophages. Furthermore, macrophages were incubated with different concentrations of INF in solution to comparatively assess the ability of the free and microencapsulated infliximab to neutralize TNF-α produced by THP-1.

After incubating WEHI13-VAR cells with supernatant from macrophages treated with incremental free INF doses, the infliximab concentration was plotted against the percentage of WEHI cell death inhibition, leading to a sigmoidal dose–response curve (see Supplementary Materials). The curve fits well with the four-parameter logistic regression model (4-PL; *R*^2^ = 0.9906), yielding an IC50 value of 49.24 ng/ml. The complete neutralization of TNF-α produced by macrophages was reached after treatment with 2.4 µg/ml of infliximab.

**Fig. 8:**
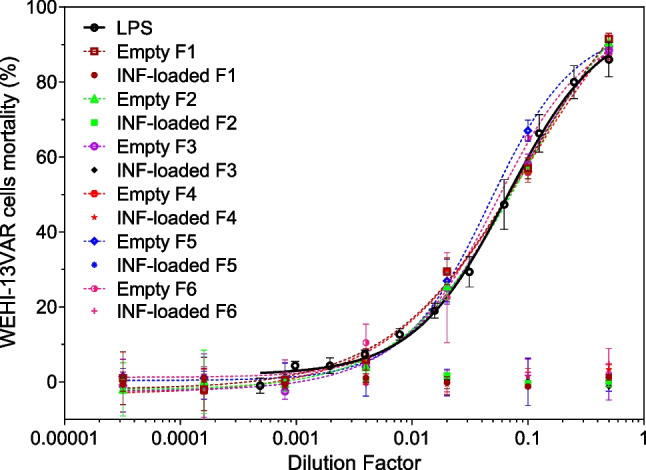
Percentage of cell death observed in WEHI-13VAR cell line after co-incubation with serial dilutions of culture medium recovery from LPS-stimulated THP-1 macrophage cell cultures in absence of treatment (LPS-positive control) or after its incubation with empty and INF-loaded MCs

As shown in Fig. 8, the incubation of WEHI cells with serial dilutions of the supernatant obtained from cultures of LPS-prestimulated THP-1 macrophages, in the absence of treatment, led to dose-dependent cytolysis of WEHI cells mediated by TNF-α. The sigmoidal curve obtained by plotting the decimal logarithm of the dilution factor against the observed percentage of WEHI cells mortality was adjusted to a logistic regression model (4-PL), estimating a value of − 1.179 for the decimal logarithm of the lethal dose 50 (LD50). Thus, a 1/15 dilution of the macrophage supernatant in our experimental conditions contains enough TNF-α to produce in vitro the 50% mortality of 50% of WEHI cells.

The effect of empty microcapsules was evaluated. Obtained results showed that treatment of LPS-stimulated macrophages with 200 µg/ml of empty MCs from the 6 formulations developed had no significant effects on TNF-α production, leading to dose–response curves that overlap with the obtained for the LPS control in the absence of treatment (Fig. 8). These results confirmed that empty MCs do not stimulate the release of TNF-α by THP-1 macrophages in vitro, suggesting that MCs do not exert a proinflammatory effect per se.

Regarding the effect of microencapsulated INF, incubation of THP-1 macrophages with 200 µg/ml of INF-loaded MCs led to the complete neutralization of bioactive TNF-α produced by macrophages, demonstrating that infliximab preserved its biological activity once microencapsulated. In addition, these results confirmed the high in vitro anti-inflammatory activity of INF-loaded MCs. Taking into account the theoretical load of INF in the microcapsules and its encapsulation efficiency, the total amount of microencapsulated INF incorporated into the cultures ranged from 1.4 to 5.6 µl/ml, depending on the formulation. However, even though it was impossible to monitor the release profile at the extra- and intracellular lever during in vitro cell culture assays, according to the results of the in vitro delivery assays of INF under *sink* conditions, it can be expected that maximum theoretical concentrations of INF in culture medium were not reached during the assay. Concisely, over the incubation period of formulations with THP-1 cells (6 h), it would be expected a release of INF lower than 20% of the total drug content for the larger formulations (F1, F2, F3) and lower than 50% for those prepared by emulsion evaporation, which would result approximately in INF concentrations ranging between 0.59 and 1.12 µg/ml.

Comparison of the TNF-α neutralization capacity of free INF (Fig. 8) in this concentration range with the complete neutralization observed after treatment with INF-loaded MCs suggests that the incorporation of INF into polymeric microcapsules could have given rise to an increase in the efficacy of INF in vitro. This hypothesis was reinforced in the light of the results obtained when investigating the production of TNF-α and Il-6 by THP-1 macrophages using ELISA (“TNF-α and Il-6 production”), observing a more significant suppressive effect on the production of both mediators in response to INF-loaded MCs compared to similar concentrations of the free drug. However, this hypothesis must be validated in tests in an in vivo environment and over an extended time.

The preservation of INF biological activity during the microencapsulation process, regardless of the encapsulation method used, confirms the excellent properties of PEOT-PBT copolymers for microencapsulating macromolecules without compromising their three-dimensional conformation and, therefore, their bioactivity. These properties derive from the amphiphilic character of these block copolymers, which variants with a high proportion of the soft segment, like the one used in this work, contain a majority of highly hydrophilic domains that allow hosting these macromolecules without compromising their stability. In this sense, the preparation of microparticles with these copolymers was shown to preserve the biological activity of various bioactive proteins, including lysozyme [22], a dimer of anti-VEGF monoclonal antibodies intended for intravitreal injection [20], and interferon-a2b, whose sustained release formulation based on microspheres composed of Polyactive^®^ (Locteron™) has already reached phase II of clinical development in humans for its application in the treatment of hepatitis C [21].

The results achieved after the microencapsulation of INF demonstrated that the incorporation of PLGA into the polymeric matrix, in a low proportion, allowed us to modulate several aspects of the formulations obtained, such as the encapsulation efficiency, particle size, surface hydrophobicity, or in vitro delivery profile without compromising its excellent properties in terms of preserving the activity of the encapsulated macromolecules. The promising results achieved for the INF formulations developed must be further evaluated in vivo in an animal model of RA to assess their potential clinical applications.

### Cytotoxicity evaluation

**Fig. 9:**
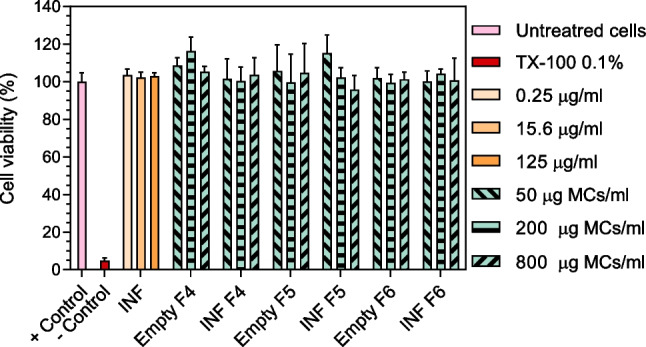
WST-1 assay. Percentage of viability of THP-1 macrophages after 24 h of incubation with the MCs obtained by Em/Ev [F4 (PEOT-PBT); F5 (65:35 PEOT-PBT:PLGA RG502); F6 (65:35 PEOT-PBT:PLGA RG503)]

**Fig. 10:**
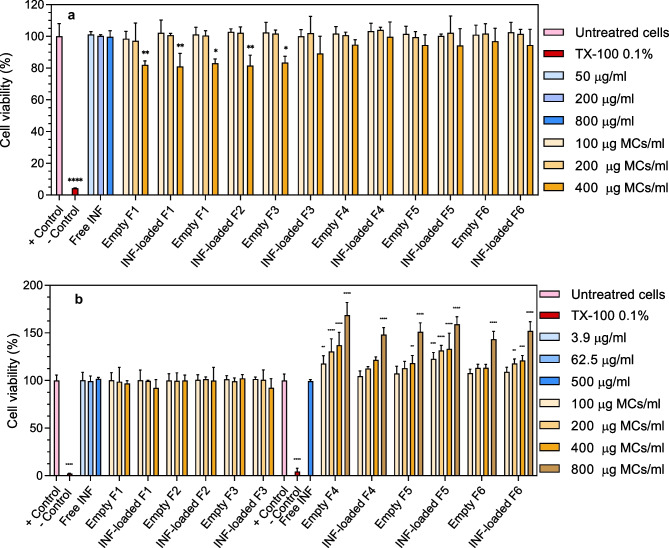
XTT **a **and Resazurin-based **b **assay. Percentage of viability of THP-1 macrophages after 24 h of incubation with the MCs. * indicates statistically significative differences with respect to positive control (untreated cells): *(*p* ≤ 0.05); **(*p* ≤ 0.01); ***(*p* ≤ 0.001); ****(*p* ≤ 0.0001)

Cell compatibility of the developed formulations was investigated in vitro in THP-1 macrophages. Briefly, differentiated cells were incubated for 24 h with different concentrations of INF-loaded MCs, empty MCs, and free infliximab. Subsequently, for comparison, the percentage of cell viability was determined by three widely used cytotoxicity assays. Concisely, WST-1 (Fig. 9), XTT (Fig. 10a), and a Resazurin-based cytotoxicity assay (Fig. 10b) were performed. Obtained results demonstrate the biocompatibility of all the formulations developed regardless of their polymeric composition and microencapsulation technique, following the results previously reported in the literature [59, 60].

Focusing on the results of the WST-1 cell proliferation assay (Fig. 9), only performed for the formulations prepared by emulsion evaporation (F4–F6), the non-parametric statistical analysis (Kruskal–Wallis) confirmed the absence of statistically significant differences (*p* > 0.05) between the viability percentage of untreated cells and cell co-incubated with different concentrations of free INF (0.25–125 µg/ml), empty MCs, and INF-loaded MCs (50–400 µg MCs/ml). These results agreed with those obtained using the XTT assay (Fig. 10a), supporting the absence of toxicity of all formulations developed (F1–F6) in the concentration interval investigated. However, statistical analysis showed significant differences in cell viability, although the post hoc analysis confirmed that the significant reduction in cell viability occurred only with the maximum dose of MCs (400 µg MCs/ml) of formulations F1, F2, and F3. This phenomenon seems to be related to a limitation inherent to the experimental conditions when high concentrations of particles are incorporated into the culture since the magnitude of the observed effect is related to the size and concentration of the microparticles, as demonstrated by the highest percentage of viability obtained after co-incubation of cells with formulations of identical composition and smaller particle size (F4–F6). Therefore, in light of these results, a maximum concentration of 200 µg MCs/ml (62.5 µg/cm^2^) was selected for subsequent in vitro characterization assays on this cell line.

Finally, to contrast the obtained results, the biocompatibility of free INF and developed formulations was investigated using an assay based on resazurin metabolization, the mechanism of the widely used AlamarBlue^®^ commercial assay. The results obtained (Fig. 10b) for free INF and the formulations prepared by UA agreed with those obtained by the XTT assay, although a smaller reduction in cell viability was observed after treatment with a concentration of 400 µg MCs/ml. Nevertheless, the results obtained for the formulations prepared by emulsion evaporation showed an increase in cell viability in a dose-dependent manner, reaching viability values higher than 150% for the maximum concentration of MCs (800 µg MCs /ml).

These results contrast with those obtained through the WST-1 and XTT assays, so they were attributed to some interference concerning the analytical determination since the results obtained were consistent after replication of the assay. To go further into the possible causes and discard a possible reduction of resazurin by some component present in the culture medium or formulations, similar concentrations of MCs were incubated with cell-free culture medium without phenol red for 24 h under culture conditions and the assay was subsequently performed following the usual protocol. The fluorescence intensity registered for the resazurin solution incubated with the formulations did not present differences with the negative control. At the same time, the direct fluorometric measurement of the plate in the presence of the MCs led to a none dose-dependent slight increase in the signal obtained, but of very low magnitude compared to the increase observed in the presence of cells. Therefore, it was not possible to elucidate the origin of the observed interaction, although everything points to the need for the cell culture’s presence for it to occur. This observation highlights the need to compare the cytocompatibility results obtained in vitro by using several cell viability assays based on different mechanisms, even when widely characterized tests used in similar investigations are used, thus avoiding the risk of getting false positives.

In conclusion, the biocompatibility of all INF-loaded formulations developed has been verified, regardless of their polymeric composition and the microencapsulation technique used in their preparation. In addition, treatment of macrophages with a wide concentration range of INF in solution (0.25–800 µg/m) did not produce toxic effects on THP-1 cells.

### Phagocytic capacity of THP-1-derived macrophages

The ability of the developed formulations to be internalized by macrophages was investigated using fluorescent microcapsules labelled with Coumarin 6. In brief, THP-1 monocytes differentiated into macrophages were incubated with different doses of microcapsules for 2.5 h, and, subsequently, the percentage of phagocytosed particles was quantified through a fluorimetric assay. Additionally, to further characterize the extent of the phagocytic process and the particle size preferentially internalized by macrophages, a fluorescent nuclear stain was applied to the cultures, and they were observed by fluorescence light microscopy.

**Fig. 11:**
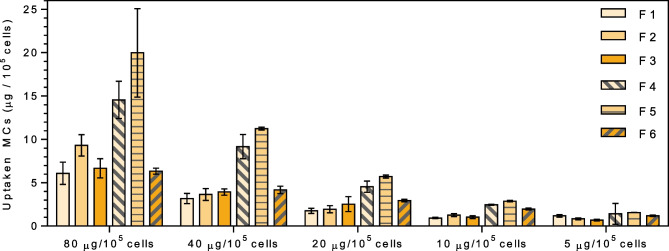
Micrograms of fluorescent-labelled MCs internalized by THP-1 macrophages after its incubation for 2.5 h with different concentrations (5–80 µg/10.^5^ cells) of MCs obtained by UA [F1 (PEOT-PBT); F2 (65:35 PEOT-PBT:PLGA RG502); F3 (65:35 PEOT-PBT:PLGA RG503)] and Em/Ev [F4 (PEOT-PBT); F5 (65:35 PEOT-PBT:PLGA RG502); F6 (65:35 PEOT-PBT:PLGA RG503)]

The obtained results showed that all the formulations are composed of particles with adequate properties to be engulfed by macrophages and achieve a potential intracellular drug release. All formulations were extensively internalized by phagocytes in a dose-dependent manner (Fig. 11), although differences were observed in the degree of internalization of the different microparticle formulations. Concisely, a correlation was observed between the percentage of phagocytosis and the mean particle size of the formulations, which increased as the mean diameter of the MCs decreased. Therefore, except for F6, MCs prepared by emulsion evaporation showed a higher degree of internalization than those with an identical composition prepared by ultrasonic atomization, which can almost certainly be attributed to the influence of the microencapsulation technique on the particle size, which is one of the key factors in the phagocytic process [61].

Also, the influence of microparticle hydrophobicity and surface charge on the extension of the phagocytic process is well known, being preferentially internalized the hydrophobic particles with a higher absolute value of ζ potential [62]. However, although it was impossible to establish a direct correlation between surface hydrophobicity and the percentages of phagocytosis obtained, significant differences have been observed in the percentage of internalized MCs between formulations with a comparable mean size and significantly different surface hydrophobicity. Concisely, formulation F4 (2.09 ± 0.47 µm) was phagocytosed to a greater extent compared to F6 (1.71 ± 0.27 µm), which could be related to its different polymer composition. It should be noted that F6, despite their significantly lower mean particle size, showed a similar uptake to MCs fabricated by UA whit the same composition (F3), which may be explained by the higher aggregation degree observed for F6 in culture medium when performed experiments. As well known, higher size of aggregates compared to single particles and its irregular surface could decrease the internalization extent. This hypothesis is reinforced by the observation of no significant differences between the internalization rate of larger F1 and F3 MCs, whit negligible aggregation degree, although their analogous composition to F4 and F6, respectively. Also, this behavior may result from the larger mean size of the formulations prepared by UA due to a limitation in the amount of particles available within the phagocytable range. In this context, the MCs prepared by UA exhibit a polydisperse distribution so that only a fraction of the formulation has an optimal size to be phagocytosed. As a consequence, those formulations present the ideal properties for achieving the combined intracellular and extracellular release of infliximab and retention of the release system in the joint cavity for long periods, so particle size has been identified as a critical factor in achieving effective retention of the particles in the inflamed joint [15].

**Fig. 12:**
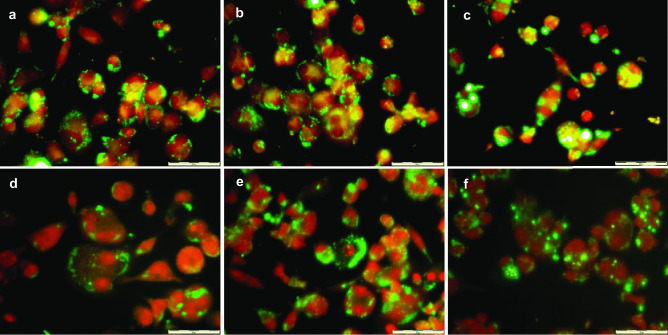
UV microscopy photomicrographs showing the fluorescent MCs phagocytosed by THP-1 macrophages: **a** F1 (UA; PEOT-PBT); **b** F2 (UA; 65:35 PEOT-PBT:PLGA RG502); **c** F3 (UA; 65:35 PEOT-PBT:PLGA RG503); **d** F4 (Em/Ev; PEOT-PBT), **e** F5 (Em/Ev; 65:35 PEOT-PBT:PLGA RG502), and **f** F6 (Em/Ev; 65:35 PEOT-PBT:PLGA RG503). The scale bar represents 50 µm

Microscopic analysis of cell cultures co-incubated with MCs (Fig. 12) confirmed the high phagocytic activity of the macrophages over the formulations. Likewise, small, non-aggregated, spherical particles inside the macrophages were clearly observed, with a high mean number of particles per cell. The assessment of phagocytosis in terms of the phagocytosis index, expressed as the percentage of cells that phagocytosed at least one particle, was higher than 98% for all the investigated formulations.

Otherwise, intending to assess the potential for an intracellular release of infliximab, it is not possible to know exactly how the intracellular environment of the particles, once phagocyted, could affect the stability of INF. Due to the extreme complexity of the phagocytic process, which may involve phagosome fusion with lysosomes and the consequent exposure of phagocytic material to highly acidic pH and numerous degradative enzymes, it is not foreseeable that monoclonal antibody stability can be maintained at the intracellular level long term. Nevertheless, the development of systems intended for the intracellular delivery of monoclonal antibodies has generated interest in the last decade, emerging some systems, such as lipidic or polymeric particles, which could protect the monoclonal antibody against the degradative environment at the intracellular level, thus avoiding its premature degradation [63, 64]. Concisely, intracellular delivery of bevacizumab-loaded polymeric nanoparticles was achieved [65].

Despite how phagocytosis could have contributed to the observed therapeutical effect could not be determined, the obtained results after treatment of macrophages with INF-loaded MCs in vitro (“TNF-α and Il-6 production”) confirmed the existence of a marked acute anti-inflammatory effect, leading to a significant decrease in the levels of TNF-α and Il-6, demonstrating the promising potential of this type of formulations.

### TNF-α and Il-6 production

The anti-TNF-α monoclonal antibody infliximab is a biological DMARD that, like other anti-TNF-α, has shown high efficacy in inducing and maintaining clinical remission and low disease activity in rheumatoid arthritis [1]. In the context of developing a microparticle formulation intended for the intra-articular release of INF, it is interesting to investigate the effect of INF-loaded MCs on the neutralization and production of TNF-α and other proinflammatory mediators (such as Il-6) by phagocytic cells. That is especially important considering that activated macrophages are present in the arthritic joint cavity and play a pivotal role in RA as the main amplifiers of inflammation [66, 67].

**Fig. 13:**
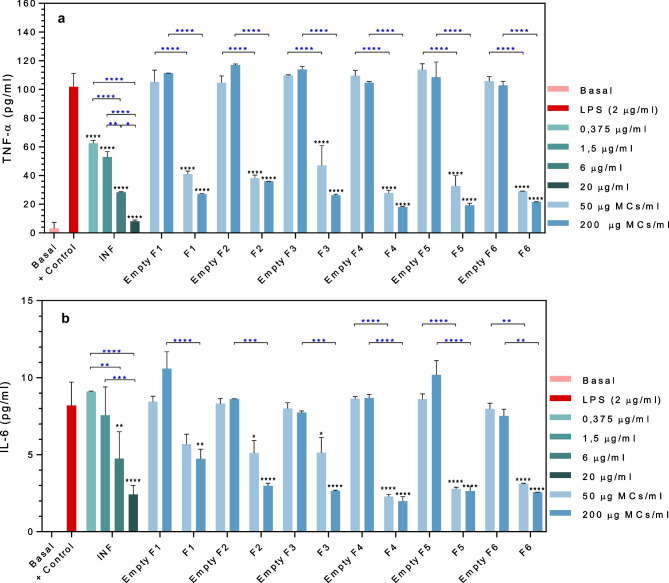
TNF-α **a **and Il-6 **b **production by LPS-stimulated THP-1 macrophages in response to treatment with free and microencapsulated INF by UA [F1 (PEOT-PBT); F2 (65:35 PEOT-PBT:PLGA RG502); F3 (65:35 PEOT-PBT:PLGA RG503)] and Em/Ev [F4 (PEOT-PBT); F5 (65:35 PEOT-PBT:PLGA RG502); F6 (65:35 PEOT-PBT:PLGA RG503)]. TNF-α and Il-6 concentrations in culture medium were measured by ELISA. *(*p* ≤ 0.05); **(*p* ≤ 0.01); ***(*p* ≤ 0.001); ****(*p* ≤ 0.0001)

The levels of TNF-α and Il-6 produced in vitro by THP-1 macrophages prestimulated with LPS in response to its treatment with naïve and INF-loaded MCs were investigated to evaluate the potential anti-inflammatory effect of the developed formulations. Inflammatory mediators were quantified using enzyme immunoassays (ELISA) specific for human TNF-α and Il-6, according to the calibration curves obtained from recombinant standards for each analyte. The plot between the decimal logarithm of the TNF-α concentration and the absorbance (*λ* = 450 nm) led to a symmetric sigmoidal curve fitted to a 4-PL regression model (*R*^2^ = 0.9997; adj-*R*^2^ = 0.9993). Regarding the Il-6 calibration curve, the relationship between concentration and absorbance fitted a second-degree polynomial function (*R*^2^ = 0.9999; *R*^2^ = 0.9999). Figure 13 summarizes the levels of TNF-α and Il-6 produced by macrophages in response to the different treatments and conditions assayed, as well as the results of the analysis of variance (ANOVA) and post hoc analysis by Tuckey’s test.

Treatment of macrophages in vitro with INF-loaded MCs led to a statistically significant decrease in TNF-α and Il-6 levels compared to the LPS-positive control. Additionally, a slight dose-dependent reduction in TNF-α levels was observed after treatment with 200 and 50 µg/ml of INF-loaded MCs, although no statistically significant differences were reached between both doses. The differences observed between the six INF formulations investigated gave rise to a statistically significant effect (Fig. 13a). However, the formulations prepared by emulsion evaporation (F4, F5, and F6) exerted a slightly higher effect, surprising considering their low encapsulation efficiency compared to those prepared by UA. This effect might be due to the combination of several factors: 1. The higher in vitro INF release found for these formulations and 2. The smaller particle size distribution is a well-known parameter that significantly affects cell-particle interactions, such as phagocytosis or the uptake rate of particles.

Obtained results for Il-6 production by macrophages after exposure to microencapsulated INF followed the trend observed for TNF-α levels, reaching a statistically significant decrease in interleukin- 6 production in response to all formulations assayed. The correlation between TNF-α and Il-6 reduction may be explained due to the higher position of TNF-α within the hierarchical chain of cytokine production, so Il-6 production depends directly on the stimulation by TNF-α. This hypothesis is reinforced by taking into account that the mechanism of action of INF as a therapeutical antibody is its high affinity binding to TNF-α, thus preventing it from exerting its biological effect. However, it should be noted that a dose-dependent effect of greater magnitude was observed for Il-6 compared to TNF-α, especially in response to the three formulations prepared by ultrasonic atomization. In addition, incubating the macrophages with the higher doses of empty MCs from all formulations developed did not lead to a statistically significant change in the concentration of both investigated mediators.

To further investigate the effect of the INF formulations, macrophages were also treated with free INF in solution at concentrations comparable to those theoretically obtained after the complete release of the microencapsulated antibody. Table 4 displays these theoretical concentrations for each formulation and dose investigated to facilitate the interpretation of the result.

**Table 4:** Theoretical concentration of INF reached in culture medium after complete delivery of INF from the microcapsules for both concentrations of MPs assayed (200 y 50 µg MCs/ml)

	**INF concentration (µg/ml)**			**INF concentration (µg/ml)**	
	**200 µg MCs/ml**	**50 µg MCs/ml**		**200 µg MCs/ml**	**50 µg MCs/ml**
**F1**	4.88 µg/ml	1.22 µg/ml	**F4**	1.84 µg/ml	0.46 µg/ml
**F2**	4.92 µg/ml	1.23 µg/ml	**F5**	1.38 µg/ml	0.35 µg/ml
**F3**	5.61 µg/ml	1.40 µg/ml	**F6**	1.70 µg/ml	0.43 µg/ml

Treatment of macrophages with free INF (0.35–20 µg/ml) caused a statistically significant reduction in TNF-α production in a dose-dependent manner. The same effect was observed on Il-6, although a statistically significant reduction of Il-6 production in vitro was only achieved in response to doses higher or equal to 6 µg/ml. The comparison of the results obtained for equivalent doses of INF, free and incorporated in the MCs, showed that the microencapsulated INF had a significantly stronger effect than the free drug, being the reduction over Il-6 production more significant than the observed for TNF-α. Furthermore, this phenomenon was more pronounced for the treatment with low doses of MCs, regardless of their polymeric composition and microencapsulation technique. For example, treatment with 1.5 µg/ml of free INF decreased, respectively, 48.04% and 7.59% the production of TNF-α and Il-6. However, treatment with 50 µg MCs/ml, equivalent to an INF concentration of 1.22 (F1), 1.23 (F2), and 1.40 µg/ml (F3), caused, respectively, a significative reduction in TNF-α (59.7%, 62.6%, 53.6%) and Il-6 (42.2%, 63.6%, and 67.6%) production levels.

These results unequivocally confirm that the INF preserved its biological activity once microencapsulated and the marked anti-inflammatory effect in vitro of all INF-loaded microparticulate formulations developed. Furthermore, INF microencapsulation resulted in a significant increase in the ability of INF to neutralize the produced TNF-α and the consequent reduction in Il-6 production. This finding confirmed the results of the WEHI cell bioactivity assay.

The causes underlying the higher in vitro anti-inflammatory activity observed for microencapsulated INF could not be elucidated. This observation probably responds to the interaction of multiple factors, ranging from the pleiotropic mechanism of INF, which in addition to blocking soluble TNF-α can bind to transmembrane TNF-α, modifying the cell signalling process in a complex way, to others such as particle phagocytosis and the potential intracellular release of INF, whose potential advantages were previously discussed [64, 65]. On the other hand, MCs could protect INF from degradation and aggregation in a cell culture medium, which could decrease its activity when it is in solution. In any case, elucidating these causes would be of great interest in the later stages of this investigation and requires the detailed performance of additional specific assays, which go beyond the objectives initially set for this work.

The high anti-inflammatory effect observed in vitro for all INF formulations developed, in combination with their sustained release profile and their potential to be effectively retained in the joint cavity, with greater retention being expected for larger MCs, demonstrate the promising potential of these formulations to achieve an effective local administration of infliximab at the intra-articular level.

## Conclusions

Infliximab microencapsulation was successfully developed using Polyactive^®^ 1000PEOT70PBT30 and its blends with PLGA RG502 and RG503 (PEOT-PBT:PLGA; 65:35) as a biodegradable polymeric matrix. Two encapsulation methods (UA and Em/Ev) were compared, obtaining six formulations of spherical core–shell INF-loaded microcapsules. The UA technique proved highly effective in encapsulating INF, with loading efficiency values ranging from 69.7 to 80.25%, while emulsion/evaporation showed lower values (17.3 to 23.0%). Regarding particle size distribution, emulsion/evaporation outperformed UA, yielding a smaller mean particle size (1.5–2.1 µm) and narrower distribution than ultrasonic atomization (26.6–49.9 µm). Nonetheless, those differences in particle size would allow us to achieve different retention times in the inflamed joint when administered intra-articular, with potential higher retention for MCs prepared by UA. Sustained INF release for up to 24 days was achieved, with polymeric composition and microencapsulation technique significantly influencing delivery profiles. Notably, the MCs prepared by Em/Ev showed a higher initial INF release than those obtained through UA, followed by a sustained delivery in all formulations, whit lower release rates for PEOT-PBT compared to PEOT-PBT: PLGA blends. Regardless of the polymeric matrix and encapsulation method, microencapsulated INF preserved its structural integrity and biological activity, effectively neutralizing TNF-α production and exhibiting higher efficacy in vitro than free INF at comparable doses. All formulations were biocompatible and extensively phagocyted by THP-1 macrophages. INF-loaded MCs exhibited significative anti-inflammatory activity in vitro regarding TNF-α and Il-6 production, with improved behavior compared to commercialized INF in solution. In conclusion, the developed INF-loaded microcapsules show promising potential for long-term intra-articular delivery. However, further in vivo studies are needed to confirm this and determine the therapeutic INF doses and dosing intervals for optimal joint cavity treatment.

### Supplementary Information

Below is the link to the electronic supplementary material.

Supplementary file1 (DOCX 2645 KB)

## Data Availability

The datasets generated during and/or analyzed during the current study are available from the corresponding author on reasonable request.
