# Supplementary material for: Infliximab microencapsulation: an innovative approach for intra-articular administration of biologics in the management of rheumatoid arthritis—in vitro evaluation
Source: Drug Deliv Transl Res. 2023 Jun 9;13(12):3030–58. doi: 10.1007/s13346-023-01372-1 (PMC10624745; doi:10.1007/s13346-023-01372-1)
Supplement: Supplementary file 1 — Supplementary file1 (DOCX 2645 KB) [file 13346_2023_1372_MOESM1_ESM.docx]

Supplementary to section 3.1: Infliximab microencapsulation

**Table 2.** Process yield (P.Y) and Encapsulation Efficiency (E.E) of developed formulations.

|  | **PEOT-PBT** | **PLGA RG502** | **PLGA RG503** | **Technique** | **P.Y. (%)** | **E.E (%)** |
| --- | --- | --- | --- | --- | --- | --- |
| **F1** | 100 % | 0 % | 0 % | UA | 84.2 ± 2.1 | 69.74 ± 3.21 |
| **F2** | 65 % | 35 % | 0 % | UA | 94.5 ± 1.9 | 70.32 ± 3.18 |
| **F3** | 65 % | 0 % | 35 % | UA | 91.3 ± 1.7 | 80.21 ± 2.97 |
| **F4** | 100 % | 0 % | 0 % | Em/Ev | 68.7 ± 2.9 | 22.99 ± 1.90 |
| **F5** | 65 % | 35 % | 0 % | Em/Ev | 84.1 ± 3.2 | 17.26 ± 0.92 |
| **F6** | 65 % | 0 % | 35 % | Em/Ev | 79.2 ± 3.1 | 21.33 ± 1.02 |

Supplementary to section 3.5: Surface Hydrophobicity

**Fig. s1** Langmuir (a) and Freundlich (b) adsorption isotherms of Rose Bengal to the surface of microcapsules fabricated by UA in PBS 100 mM, pH 7.4 at 20ºC: F1 (PEOT-PBT), F2 (65:35 PEOT-PBT: PLGA RG502) and F3 (65:35 PEOT-PBT: PLGA RG503).

Supplementary to section 3.6: Differential Scanning Calorimetry

**Fig. S2.** DSC thermogram of INF (Remicade^®^) in solution (PBS 10 mM, pH 7,2). Characteristic endothermic transitions attributed to the denatured variable domain of the protein (F_ab_) and the CH_3_ domain of the heavy chains were detected.

**Fig. S3** DSC thermograms of microparticulate formulations fabricated by UA: Empty F1 (a), F1 (b), Empty F2 (c), F2 (d), Empty F3 (e), F3 (f).

Supplementary to section 3.7: Fourier Transform Infrared Spectroscopy


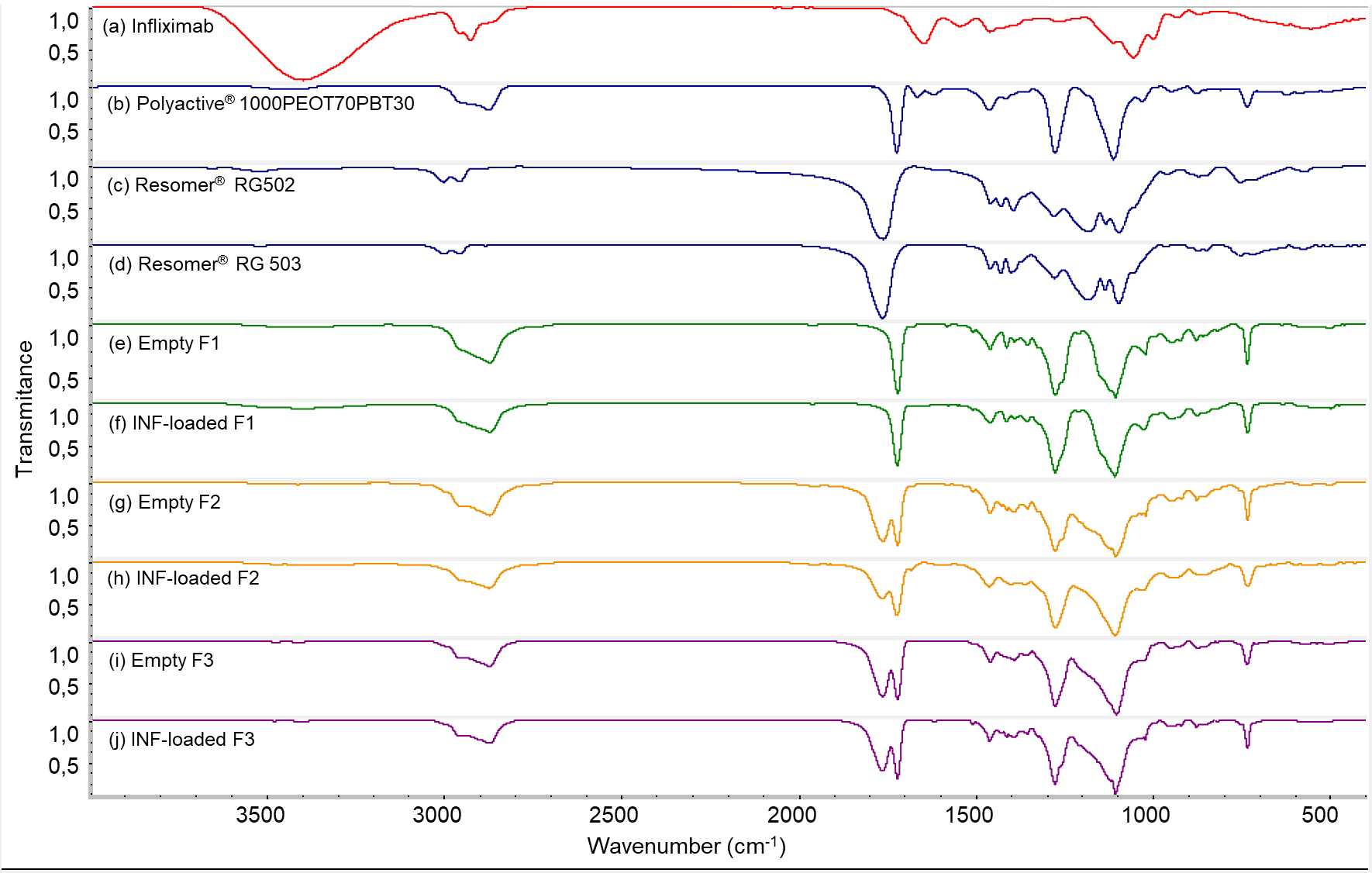


**Fig. S5** FTIR spectra of pure Infliximab (a), Polyactive^®^ 1000PEOT70PBT30 (b), Resomer^®^ RG502 (c), Resomer^®^ RG503 (d), empty MCs (e, g, i) and INF-loaded MCs (f, h, j).

**Fig. S4** DSC thermograms of Polyactive^®^ 1000PEOT70PBT30 (a), Resomer^®^ RG502 (b), Resomer^®^ RG503 (c), Empty F4 (d), F4 (e), Empty F5 (f), F5 (g), Empty F6 (h), F6 (i).

Supplementary to section 3.9: Structural stability of infliximab


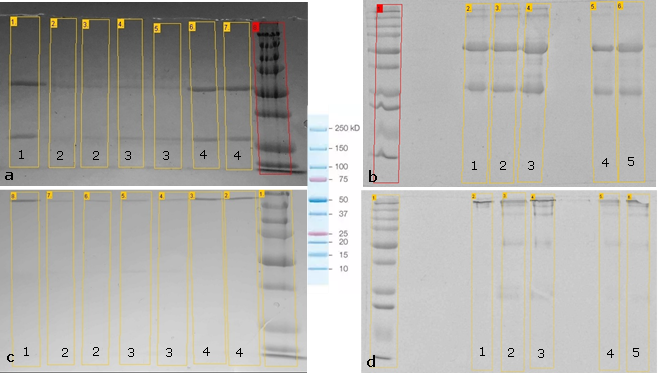


**Fig. S6** SDS page electrophoresis under reducing (a, b) and non-reducing (c, d) conditions of INF standard (1a, 5b; 1c, 5d), Ultrasonic atomized infliximab (4b; 4d) and INF extracted from microparticulate formulations F1(1b; 1d), F2 (b2; d2),, F3 (b3; d3), F4 (a2, c2), F5 (a3, c3) and F6 (a4, c4).

Supplementary to section 3.10: Biological activity of microencapsulated INF

**Fig.S7** Dose-response curve obtained for the neutralization of TNF-α produced by THP-1 macrophages after incubation with different concentrations of INF in solution.
